# Cyclin-dependent kinase 7 is a therapeutic target in high-grade glioma

**DOI:** 10.1038/oncsis.2017.33

**Published:** 2017-05-15

**Authors:** S A Greenall, Y C Lim, C B Mitchell, K S Ensbey, B W Stringer, A L Wilding, G M O'Neill, K L McDonald, D J Gough, B W Day, T G Johns

**Affiliations:** 1 Centre for Cancer Research, Hudson Institute of Medical Research, Clayton, Victoria, Australia; 2 Brain Cancer Discovery Collaborative, Australia; 3 Monash University, Clayton, Victoria, Australia; 4 Translational Brain Cancer Research Laboratory, QIMR Berghofer Medical Research Institute, Herston, Queensland, Australia; 5 Focal Adhesion Biology Group, Children’s Hospital at Westmead, Westmead, New South Wales, Australia; 6 Cure Brain Cancer Neuro-Oncology Laboratory, Prince of Wales Clinical School, University of New South Wales, Sydney, New South Wales, Australia

## Abstract

High-grade glioma (HGG) is an incurable brain cancer. The transcriptomes of cells within HGG tumors are highly heterogeneous. This renders the tumors unresponsive or able to adapt to therapeutics targeted at single pathways, thereby causing treatment failure. To overcome this, we focused on cyclin-dependent kinase 7 (CDK7), a ubiquitously expressed molecule involved in two major drivers of HGG pathogenesis: cell cycle progression and RNA polymerase-II-based transcription. We tested the activity of THZ1, an irreversible CDK7 inhibitor, on patient-derived primary HGG cell lines and *ex vivo* HGG patient tissue slices, using proliferation assays, microarray analysis, high-resolution respirometry, cell cycle analysis and *in vivo* tumor orthografts. The cellular processes affected by CDK7 inhibition were analyzed by reverse transcriptase–quantitative PCR, western blot, flow cytometry and immunofluorescence. THZ1 perturbed the transcriptome and disabled CDK activation, leading to cell cycle arrest at G2 and DNA damage. THZ1 halted transcription of the nuclear-encoded mitochondrial ribosomal genes, reducing mitochondrial translation and oxidative respiration. It also inhibited the expression of receptor tyrosine kinases such as epidermal growth factor receptor (EGFR) and platelet-derived growth factor receptor-α (PDGFR-α), reducing signaling flux through the AKT, extracellular-signal-regulated kinase 1/2 (ERK1/2) and signal transducer and activator of transcription 3 (STAT3) downstream pathways. Finally, THZ1 disrupted nucleolar, Cajal body and nuclear speckle formation, resulting in reduced cytosolic translation and malfunction of the spliceosome and thus leading to aberrant mRNA processing. These findings indicate that CDK7 is crucial for gliomagenesis, validate CDK7 as a therapeutic target and provide new insight into the cellular processes that are affected by THZ1 and induce antitumor activity.

## Introduction

No effective therapy exists for high-grade glioma (HGG), an incurable brain cancer. Numerous advanced small molecule inhibitors and antibodies have now failed in clinical trials. HGG tumors have a high degree of intratumoral transcriptional heterogeneity, as shown by RNA sequencing of single tumor cells, and this has been implicated as a leading cause of resistance to both targeted and broad spectrum chemotherapeutics.

Single cells within HGG tumors mosaically express druggable targets from a severely altered somatic profile, usually in a mutually exclusive manner.^1, 2^ This generates sub-populations of cells within a tumor that are unlikely to respond to single agents. The isolation of single cells from an HGG tumor mass followed by their clonal expansion has revealed that each clone has a highly variable proliferative response to a panel of dozens of clinically utilized chemotherapeutics.^3^ Subsequent RNA sequencing analysis has shown that the transcriptomes of drug-resistant clones are markedly different from those of drug-sensitive clones.^3^ The plasticity of HGG transcription also allows rapid rewiring of the transcriptome to resist targeted therapeutics.^4^ This is also observed as a substantial divergence in the landscape of HGG driver alterations between tumors before patient treatment and distant recurrent tumors after treatment.^5^ The heterogeneous and adaptable transcriptomes of single HGG cells make it exceedingly difficult to kill the entire tumor and prevent recurrence.

All protein-coding genes in eukaryotic cells are transcribed by the enzyme RNA polymerase II (POLR2A). The main enzyme responsible for activating and stabilizing POLR2A during the pre-initiation phase of transcription is cyclin-dependent kinase 7 (CDK7). This ubiquitously expressed serine/threonine kinase phosphorylates serines in the carboxy-terminal domain of POLR2A, an essential step for efficient transcription.^6, 7^ Furthermore, CDK7, in complex with cyclin H and Mat1, also forms CDK-activating kinase,^8, 9^ which phosphorylates the gatekeepers of cell cycle progression (CDK1, CDK2, CDK4 and CDK6) in a process required for correct cell cycle function. The cell cycle is a prominent driver of HGG pathogenesis, with 86% of patient tumors containing at least one aberration in this pathway,^10^ resulting in unrestrained cell cycle activation. Hence, targeting CDK7 offers an attractive and untested opportunity to simultaneously inhibit two major drivers of HGG pathogenesis: RNA transcriptional diversity and cell cycle progression.

Recently, THZ1, a potent irreversible inhibitor that is selective for CDK7, was identified and has been evaluated against blood cancers, neuroblastoma, small-cell lung carcinoma, triple-negative breast cancer and esophageal squamous cell carcinoma.^11, 12, 13, 14, 15^ These studies include bioinformatics analyses on the effects of THZ1 on transcription profiles driven from super enhancers; however, there is almost no information about how THZ1 induces tumor cell death by inhibiting CDK7. In this study, we evaluated THZ1-mediated CDK7 inhibition as a potential therapeutic option for HGG. We used a large panel of clinically relevant, serum-free patient-derived primary HGG cell lines, which closely recapitulate the genotype and phenotype of the source tumor,^16, 17^ unlike traditional (fetal bovine serum cultured) lines.^16, 18^ More specifically, we examined THZ1’s effects on the global tumor transcriptional profile and used this information to identify the cellular processes perturbed by THZ1, which then contribute to the induction of cell death.

## Results

### THZ1 is highly effective at killing primary HGG in a caspase-redundant manner

**Figure 1:**
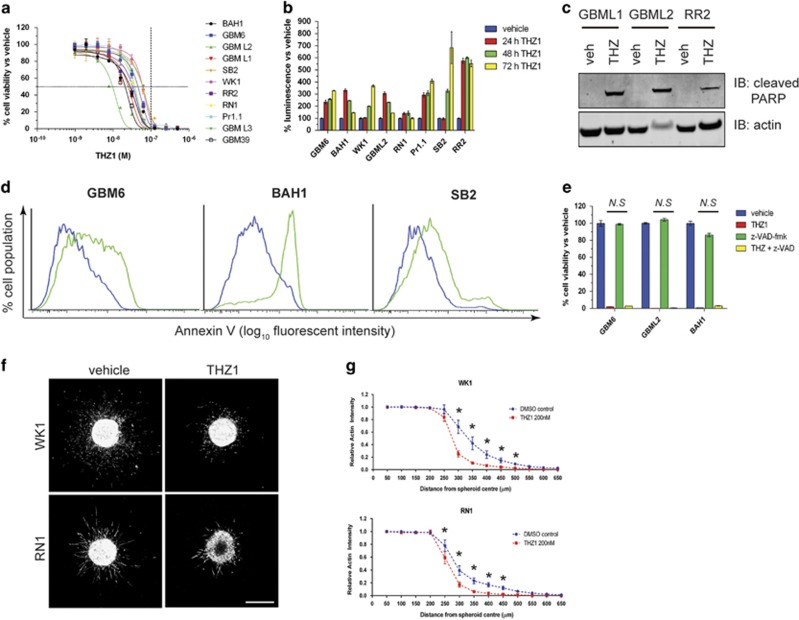
THZ1 potently inhibits gliomagenesis and prevents HGG invasion. (**a**) Eleven HGG primary cell lines, isolated from patient tissue, were treated with increasing doses of THZ1. After 7 days, cells were subjected to cell viability luminescence assays. Graph represents sigmoidal dose-response curves for all cell lines tested and their response to each THZ1 dose. Data are presented as the percentage of viable cells vs vehicle control at each dose±s.e.m. Horizontal dashed line=50% inhibition of cell viability. Vertical dashed line=100 nM THZ1 concentration. (**b**) Cleaved caspase 3/7 detection in primary cells treated with 200 nM THZ1 for 24, 48 and 72 h. Data represent the percentage luminescence signal obtained for each time point vs vehicle controls±s.e.m. (**c**) Lysates from primary cells treated for 72 h with 200 nM THZ1 analyzed for the presence of cleaved PARP (top panel) by western blotting. Actin (bottom panel) was used as a loading control. (**d**) Flow cytometry histograms for Annexin V cell-surface detection on multiple primary cell lines treated with vehicle (blue) or 200 nM THZ1 (green) for 72 h. (**e**) Cell viability assay plots for primary cell lines treated with vehicle, 200 nM THZ1, 25 μM of the pan-caspase inhibitor z-VAD-fmk or a combination of both for 7 days. Data are presented as the percentage of viable cells vs vehicle control±s.e.m. THZ1 alone vs THZ1+z-VAD-fmk, not significant (*N.S*) by two-way analysis of variance (ANOVA), d.f.=34. (**f**) Representative maximum projection confocal images of WK1 spheroids (top panels) and RN1 spheroids (bottom panels) embedded in collagen and stained for actin, after 48 h. Treatments with vehicle (left panels) or 200 nM THZ1 (right panels) are indicated. Scale bar: 500 μm (**g**) Quantification of spheroid invasion, measured by relative actin intensity over the distance from the spheroid center±s.e.m. Vehicle (dimethyl sulfoxide, DMSO) vs THZ1 for both WK1 and RN1: *P<0.0001 by two-way ANOVA. Data are the average from three independent experiments, with 8–14 individual spheroids analyzed per treatment. All experiments were repeated a minimum of two times over multiple primary cell lines.

To investigate the effect of CDK7 inhibition on HGG, we screened 11 patient-derived primary HGG cell lines that closely recapitulate a broad range of phenotypes (Supplementary Figure S1a) and genotypes (Supplementary Table S1) of HGG patient tumors for response to THZ1. THZ1 potently killed primary HGG cells, with an IC_50_<100 nM on all cell lines tested (Figure 1a) and induced apoptosis, as shown by induction of cleaved caspase 3/7 (Figure 1b), cleaved PARP (Figure 1c) and cell-surface binding of Annexin V (Figure 1d). However, co-administering the pan-caspase inhibitor z-VAD-fmk, did not protect against cell death (Figure 1e), despite this inhibitor being fully active (Supplementary Figure S1b), suggesting that THZ1 also kills cells by caspase-redundant mechanisms. When THZ1 was administered to normal immortalized BJ fibroblasts and HS5 bone marrow stromal cells, cell death occurred after 7 days of incubation but not 3 days (Supplementary Figures S1c–e). To uncover whether THZ1 is more active against proliferating cells, we seeded the HGG cell line SF767 glioma at an increasing density and incubated the cells with 200 nM THZ1 for 7 days. We observed that at very high confluence, where the cells are no longer proliferating or are proliferating very slowly, THZ1 becomes progressively less effective (Supplementary Figure S1f). Finally, THZ1 significantly retarded three-dimensional invasion of HGG cells into an extracellular matrix environment (Figures 1f and g). Collectively, these data show that CDK7 is crucial for HGG survival and that THZ1 kills proliferative HGG cells in a caspase-redundant manner while inhibiting invasion.

### THZ1 induces mitotic arrest through a G2 cell cycle block, resulting in nuclear DNA damage

**Figure 2:**
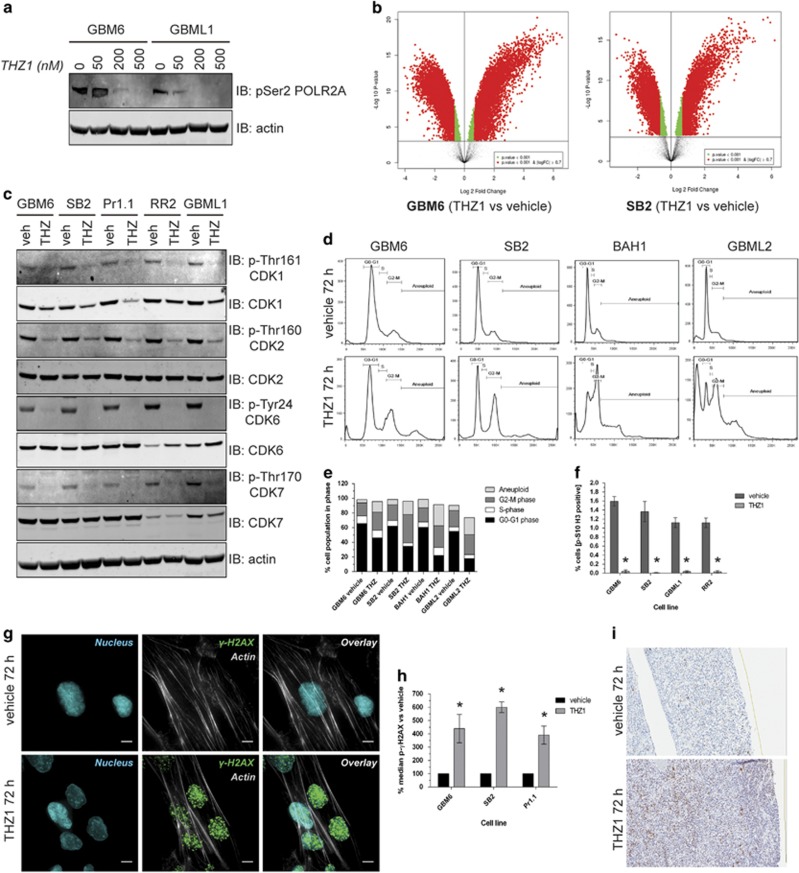
THZ1 perturbs the transcriptome and cell cycle, causing mitotic arrest at G2 phase and DNA damage. (**a**) Lysates from cells treated with increasing doses of THZ1 for 48 h analyzed for pSer2 POL2RA (top panel) by western blotting, with actin used as a loading control (bottom panel). (**b**) Volcano plots of microarray data documenting the differential expression of genes in THZ1-treated cells vs vehicle-treated cells in GBM6 (top graph) and SB2 (bottom graph). Data are presented as a log_2_ fold change in gene expression against the log_10_ *P*-value statistical probability of this change. Changes of log_2_ >0.7 with a *P*-value<0.001 are depicted in red, whereas changes of log_2_ <0.7 with a *P*-value<0.001 are depicted in green. Genes displaying no significant differential expression are depicted in black. (**c**) Lysates from cells treated with vehicle or 200 nM THZ1 for 48 h analyzed for the activation status of multiple CDKs as indicated to the right of panels by western blotting. (**d**) Histograms of four neurosphere lines permeabilized and stained with propidium iodide after 72-h treatment with vehicle or 200 nM THZ1. The cell cycle phases G0–G1, S, G2–M and aneuploidy are marked. (**e**) Graphical representation of the percentage of cells in each cell cycle phase after 72 h of vehicle or 200 nM THZ1 treatment across four primary cell lines. Data are presented as the percentage of the total cell population in each cell cycle phase. (**f**) Quantification of pSer10-Histone 3 staining by flow cytometry for primary cells treated with vehicle or 200 nM THZ1 for 24 h. Data are represented as the percentage of the total cell population staining positive for pSer10-Histone 3±s.e.m. Vehicle vs THZ1 *P<0.05 by two-way analysis of variance (ANOVA), d.f.=14. (**g**) Representative immunofluorescent z-stack images of SB2 cells treated with vehicle or 200 nM THZ1 for 72 h and stained for actin (gray), pSer139 γH2AX (green) or nucleus (blue). Scale bar: 10 μm. (**h**) Quantification of pSer139 γH2AX staining in primary cell lines treated with vehicle or 200 nM THZ1 for 72 h. Data are presented as the median pSer132 γH2AX signal for the cell population vs vehicle controls±s.e.m. Vehicle vs THZ1 *P<0.05 by multiple *t*-test analysis with Holm–Sidak correction, d.f.=4. (**i**) *Ex vivo* patient HGG tissue slices treated with vehicle or 200 nM THZ1 for 72 h before immunohistochemical staining for pSer139 γH2AX (brown) and counterstaining for nuclei (blue). All experiments were repeated a minimum of two times over multiple primary cell lines.

Next, we sought to confirm whether the other hallmarks of CDK7 inhibition such as transcriptome downregulation and cell cycle disruption also applied to HGG. THZ1 inhibited POLR2A carboxy-terminal serine phosphorylation as previously reported^11, 12, 14^ (Figure 2a). Microarray analyses (Figure 2b and Supplementary Tables S2 and S3) of complementary DNA from THZ1-treated GBM6 or SB2 cell lines indicated substantial downregulation of transcripts compared with vehicle, in agreement with previous studies.^11, 12, 14, 15^ However, thousands of transcripts were upregulated, a unique response that had not been observed.

THZ1 also decreased the activating phosphorylation of the cell cycle checkpoint mediators CDK1, CDK2, CDK4 and CDK6, as well as CDK7 (Figure 2c). Cell cycle analysis demonstrated marked cell cycle perturbation following THZ1 treatment compared with vehicle (Figure 2d), with a prominent increase in the cell populations in G2 and M accompanied by a decrease in those in G0 and G1 (Figure 2e). The population in S was essentially unchanged. An increase in aneuploid cell populations was observed in all cell lines after THZ1 treatment, which had not previously been observed for the drug (Figure 2e). As the stage at which THZ1 arrests the cell cycle is unknown, we interrogated the progression from G2 to M by staining cells for p-Histone 3, a marker of active mitosis (Supplementary Figure S2). THZ1 almost completely abolished p-Histone 3 staining in all tested cell lines (Figure 2f). Hence, THZ1 is a mitotic arrest agent, freezing cells in the G2 phase.

The observed aneuploidy most likely arises from DNA accumulation because of continuous progression to G2 but lack of transition to M. This may result in a DNA replication logjam, which causes DNA damage. THZ1-induced DNA damage was confirmed by an increase in nuclear pSer132 γH2AX signal in THZ1-treated cells compared with vehicle (Figure 2g and Supplementary Figure S3). Flow cytometric analysis showed that this DNA damage was cumulative (Supplementary Figure S4), and quantification at 72 h showed a significant (400–600%) increase in the pSer132 γH2AX signal in multiple THZ1-treated cell lines (Figure 2h). Finally, to confirm this was not an artifact of cultured cells and that this new mechanism of THZ1 action can be recapitulated in patients, we applied THZ1 to *ex vivo* slices from freshly extracted patient HGG tissue (Figure 2i). THZ1 resulted in a marked increase in pSer132 γH2AX (14% total section area) compared with vehicle-treated tissue (3% total section area). Hence, in both primary HGG cells and in patient tissue, THZ1 kills HGG cells by inducing substantial nuclear DNA damage.

### THZ1 induces damage to the mitochondria, loss of mitochondrial translation and inhibition of oxidative respiration

**Figure 3:**
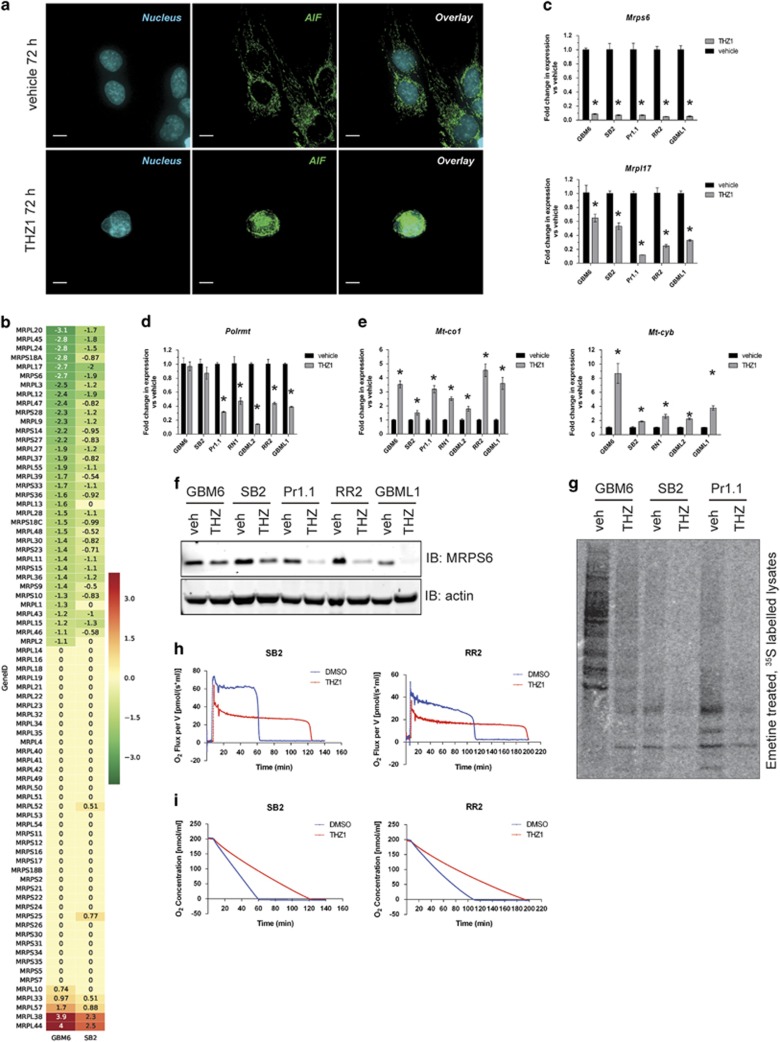
THZ1 damages mitochondria by downregulating nuclear-encoded mitochondrial ribosomal proteins, compromising mitochondrial translation and oxidative respiration. (**a**) Representative immunofluorescent z-stack images of GBM6 cells treated with vehicle or 200 nM THZ1 for 72 h and stained for apoptosis-inducing factor (AIF, green) and nucleus (blue). Scale bar: 10 μm. (**b**) Heat maps documenting the mean log_2_ expression changes of the mitochondrial ribosome subunit gene family in THZ1-treated cells vs vehicle controls in GBM6 (left column) and SB2 (right column) as measured by microarray. Green: decrease; red: increase; yellow: no change. (**c**–**e**) RT–qPCR for nuclear-encoded mitochondrial ribosomal genes *Mrps6* (**c**, top graph), *Mrpl17* (**c**, bottom graph), mitochondrial RNA polymerase *Polrmt* (**d**) and the mitochondrial-encoded genes *Mt-co1* (**e**, left graph) and *Mt-cyb* (**e**, right graph). All data are graphed as the relative expression of each gene compared with vehicle controls following correction to a multiplexed endogenous *Actb* control±s.e.m. For all graphs, vehicle vs THZ1 *P<0.05 by multiple *t*-test analysis with Holm–Sidak correction, d.f.=4. (**f**) Lysates from primary cells treated with vehicle or 200 nM THZ1 for 48 h were analyzed by western blotting for MRPS6 expression (top panel) with actin used as a loading control (bottom panel). (**g**) Autoradiographs for lysates isolated from primary cells treated with vehicle or 200 nM THZ1 for 24 h before emetine treatment followed by pulse-chase labeling for 2 h with ^35^S-labeled methionine and cysteine. (**h**, **i**) Representative plots of high-resolution respirometry conducted on live primary cells treated with vehicle or 200 nM THZ1 for 24 h. Graphs show the oxygen flux per volume over time (**h**) or the concentration of oxygen in the isolated chambers over time (**i**). All experiments were repeated a minimum of two times over multiple primary cell lines.

THZ1 may elicit other caspase-redundant mechanisms that cause DNA damage. One of these is translocation of the nuclease called apoptosis-inducing factor (AIF) from the damaged mitochondria to the nucleus, where it cleaves nuclear DNA.^19^ This mechanism was induced by THZ1 treatment, as translocation of AIF to the nucleus was observed (Figure 3a and Supplementary Figure S5a). Almost all genes responsible for mitochondrial function are nuclear encoded; therefore, we mined our microarray data (Supplementary Tables S2 and S3) and observed downregulation of half of the members of the mitochondrial ribosomal protein (MRP) family (Figure 3b). This result was confirmed by reverse transcriptase (RT)–quantitative PCR (qPCR) for two of the genes displaying the largest decreases, which were also representative of the small and large ribosomal subunit families, *Mrps6* and *Mrpl17*, across several HGG cell lines (Figure 3c). The loss of MRP transcription was reflected by a loss of MRPS6 protein following THZ1 treatment (Figure 3f). When THZ1 action was compared with the generalized transcription inhibitor actinomycin D (100 nM) and the CDK4/6 inhibitor palbociclib (1 μM) at 6 and 24 h of treatment, we saw that *Mrps6* and *Mrpl17* expression were decreased early at 6 h and even more so at 24 h with transcription inhibition but not CDK4/6 inhibition (Supplementary Figures S5b and c). Hence, THZ1 induces mitochondrial damage and induces a decrease in generalized transcription of the nuclear-encoded MRP family, resulting in mitochondrial ribosome loss.

The main transcriptional enzyme for mitochondrion-specific genes, mitochondrial RNA polymerase (*Polrmt*), is also nuclear encoded; this locus showed a significant decrease in *Polrmt* transcription (Figure 3d). Expression of other mitochondrion-specific transcription co-factors, such as *Tfam*, was largely unaffected by THZ1 at 24 h (Supplementary Figures S5d and e). Despite this, two mitochondrion-encoded genes, *Mt-co1* (Figure 3e, left panel) and *Mt-cyb* (Figure 3e, right), showed a significant increase in mitochondrial gene transcription in response to THZ1, suggesting that the loss of *Polrmt* transcription did not immediately affect mitochondrial genome transcription.

The consequences of widespread THZ1-induced loss of MRPs were confirmed by the finding that new synthesis of all mitochondrion-encoded proteins was ablated (Figure 3g), after pulsing cells with ^35^S-methionine following treatment with emetine to block cytoplasmic, but not mitochondrial, ribosomes. As these proteins are critical components of the electron transport chain, this should result in the loss of oxidative respiration. Indeed, high-resolution respirometry of THZ1-treated cells demonstrated a marked decrease in oxidative respiration rates (Figure 3h and Supplementary Figure S5f), and THZ1-treated cells more slowly depleted the finite amount of oxygen available in the sealed oxygraph chambers (Figure 3i and Supplementary Figure S5g). Hence, THZ1 potently affects the transcription of nuclear-encoded MRPs. This results in loss of electron transport chain component translation, ultimately shutting down oxidative respiration and reducing mitochondrial function.

### THZ1 is effective at downregulating the RTK/phosphatidylinositol 3 kinase/mitogen-activated protein kinase axis in HGG

**Figure 4:**
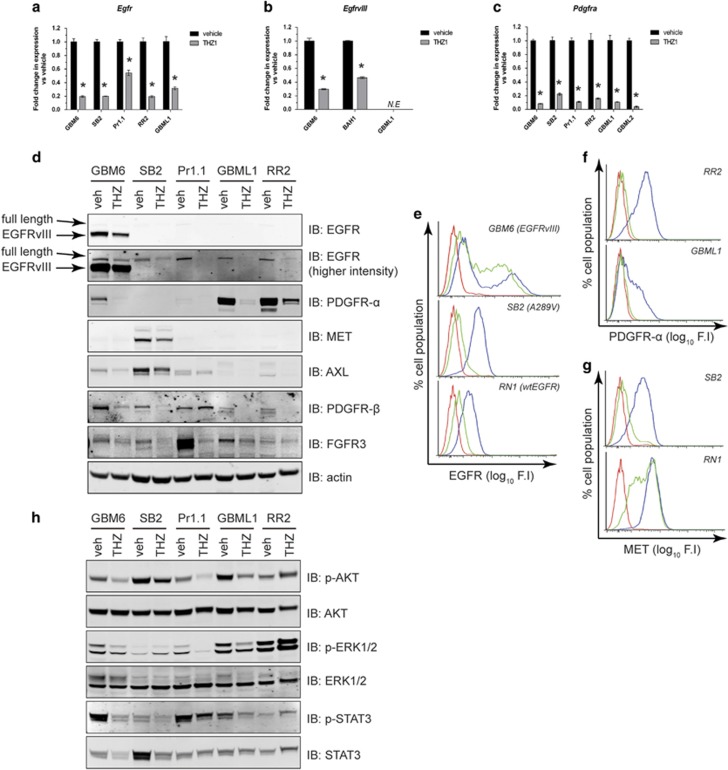
RTK expression is significantly decreased by THZ1, reducing multiple downstream oncogenic signaling fluxes. (**a**–**c**) RT–qPCR of complementary DNA isolated from primary cells treated with vehicle or 200 nM THZ1 was tested for the presence of full-length *Egfr* (GBM6, RR2 and GBML1), A289V *Egfr* (SB2) and H773_V774insPH *Egfr* (**a**), *EgfrvIII* only (**b**) or *Pdgfra* (**c**). All data are graphed as the relative expression of each gene compared with vehicle controls following correction to a multiplexed endogenous *Actb* control±s.e.m. For (**b**), the EGFRvIII-negative primary cell line GBML1 acted as a negative control. *N.E.*, not expressed. For all graphs in **a**–**c**, vehicle vs THZ1 *P<0.05 by multiple *t*-test analysis with Holm–Sidak correction, d.f.=4. (**d**) Western blot analyses of lysates from primary cells treated with vehicle or 200 nM THZ1 for 48 h for RTK protein expression. Full-length EGFR and EGFRvIII-specific bands are depicted by the arrows. Actin was used as a loading control. (**e**–**g**) Flow cytometry histograms for cell-surface expression of EGFR (EGFRvIII, wtEGFR and A289V EGFR (**e**), PDGFR-α (**f**) and MET (**g**) in multiple primary cell lines). Data are presented as percentage cell population over log_10_ fluorescence intensity of signal (F.I). Red: isotype antibody. Blue: primary antibody on vehicle-treated cells. Green: primary antibody on cells treated with 200 nM THZ1 for 48 h. (**h**) Lysates from (**d**) were analyzed for AKT, ERK1/2 and STAT3 activation by western blotting. Total AKT was used as a loading control. All experiments were repeated a minimum of two times over multiple primary cell lines.

Two important drivers of gliomagenesis, RNA transcription and the cell cycle, are therefore efficiently negated by THZ1. Another clinically important axis for gliomagenesis is the receptor tyrosine kinase (RTK)/phosphatidylinositol 3 kinase/mitogen-activated protein kinase pathway, which is perturbed in 90% of patient tumors.^10^ Further analysis of our microarray data indicated that genes encoding several important and druggable RTKs, including the epidermal growth factor receptor (*Egfr*) gene, the platelet-derived growth factor receptor-α (*Pdgfra*) gene and the *Met* gene (the most commonly altered RTKs in HGG), were expressed at lower levels in THZ1-treated cells (Supplementary Tables S2 and S3). RT–qPCR verified a loss of transcription of wild-type *Egfr*, the A289V extracellular mutant *Egfr* and the H773_V774insPH exon 20 insertion mutant *Egfr* (Figure 4a). Furthermore, *EgfrvIII*-specific RT–qPCR similarly demonstrated a marked decrease in *EgfrvIII* transcription (Figure 4b). Transcription from the *Pdgfra* locus was also significantly decreased in all HGG lines tested (Figure 4c). Hence, THZ1 negatively affects the transcription of multiple clinically relevant members of the RTK family.

At the protein level, wtEGFR, mutant EGFR and EGFRvIII expression were reduced in response to THZ1 (Figure 4d). Interrogation of other RTK family members showed decreases in PDGFR-α, MET, AXL, PDGFR-β and fibroblast growth factor receptor 3 protein expression (Figure 4d). Cell-surface expression of EGFRvIII, EGFR A289V and wtEGFR (Figure 4e), as well as PDGFR-α (Figure 4f) and MET (Figure 4g), was also significantly reduced, as demonstrated by flow cytometry. In addition, levels of p-AKT, phospho-extracellular-signal-regulated kinase 1/2 (p-ERK1/2) and phospho-signal transducer and activator of transcription 3 (p-STAT3) were reduced as shown by western blotting (Figure 4h; samples from Figure 4d). Overall, CDK7 inhibition prevents expression of the RTK family of oncogenes in HGG and reduces downstream AKT-, ERK1/2- and STAT3-based oncogenic signaling.

### THZ1 damages the nucleolar and Cajal body structures and results in loss of cytoplasmic translation

**Figure 5:**
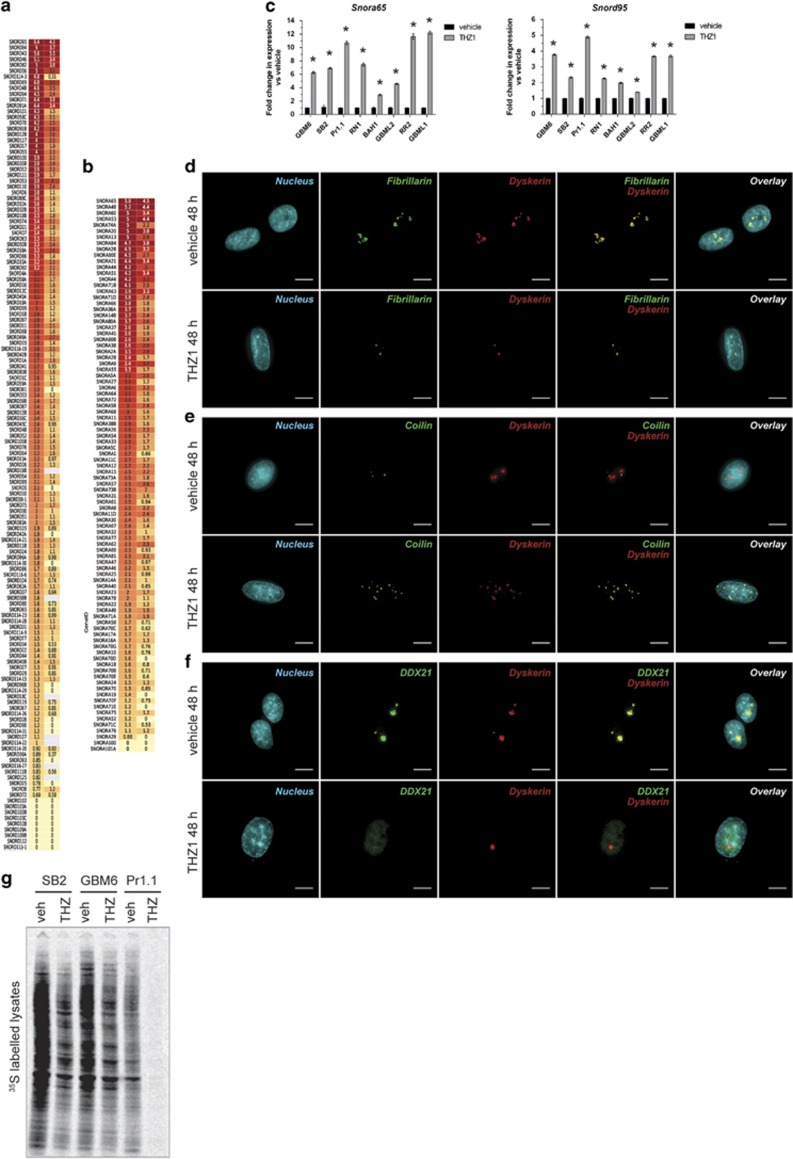
THZ1 damages the nucleolar and Cajal body structures, leading to loss of cytoplasmic translation. (**a**, **b**) Heat maps documenting the mean log_2_ expression changes of the snoRNA C/D (**a**) and snoRNA H/ACA (**b**) class of non-coding RNAs in THZ1-treated cells vs vehicle controls in GBM6 (left column) and SB2 (right column) as measured by microarray. Green: decrease; red: increase; yellow: no change. (**c**) RT–qPCR validation of upregulation of snoRNA for multiple primary cell lines treated with vehicle or 200 nM THZ1 for 24 h for *Snora65* (left graph) and *Snord95* (right graph). All data are graphed as the relative expression of each snoRNA compared with vehicle controls following correction to a multiplexed endogenous *Actb* control±s.e.m. For both graphs, vehicle vs THZ1 *P<0.05 by multiple *t*-test analysis with Holm–Sidak correction, d.f.=4. (**d**–**f**) Immunofluorescence z-stack images for GBM6 cells treated with vehicle or 200 nM THZ1 for 48 h. Images depict the nucleus in blue, dyskerin in red, fibrillarin (**d**), coilin (**e**) or DDX21 (**f**) in green, and any colocalization of signal in yellow. Scale bar: 10 μm. (**g**) Autoradiographs for lysates isolated from primary cells treated with vehicle or 200 nM THZ1 for 24 h before pulse-chase labeling for 2 h with ^35^S-labeled methionine and cysteine. All experiments were repeated a minimum of two times over multiple primary cell lines.

Our microarray results also showed that the most highly upregulated transcripts induced by THZ1 were the small nucleolar RNAs (snoRNAs) of the C/D (SNORD; Figure 5a) and H/ACA (SNORA; Figure 5b) classes. RT–qPCR confirmed the increases in *Snora65* and *Snord95* in all eight primary HGG lines tested (Figure 5c), indicating a widespread conserved response to the drug. snoRNAs are mainly concentrated in subnuclear compartments called nucleoli.^20^ Such RNAs bind to nascent ribosomal RNA (rRNA) transcripts and recruit fibrillarin (a 2′-O-methyltransferase) and dyskerin (a pseudouridylase), enabling post-transcriptional modification and stabilization of rRNA before ribosome assembly and export to the cytoplasm.^20^ snoRNAs can also be found in Cajal bodies, which are associated with spliceosome maturation.

The steep rise in snoRNA expression may indicate that THZ1 alters subnuclear structures housing these RNAs. To investigate this, we used multiplex immunofluorescence to visualize the nucleolus (using fibrillarin and dyskerin) (Figure 5d and Supplementary Figure S6a) and the Cajal bodies (using coilin) (Figure 5e and Supplementary Figure S6b). Upon THZ1 treatment, staining switched from two to four large clusters of fibrillarin and dyskerin per nucleus (Figure 5d, top) to a disrupted focally intense pattern of one to two foci per nucleus (Figure 5d, bottom) or numerous scattered foci per nucleus (Figure 5e, bottom, dyskerin). However, fibrillarin and dyskerin remained colocalized (merge, yellow signal). After THZ1 treatment, coilin staining displayed the same pattern of disruption as the nucleolus, with multiple intense foci observed (Figure 5e, bottom). Coilin also precisely colocalized with dyskerin (merge, yellow) (Figure 5e, bottom) in contrast to vehicle controls, where coilin and dyskerin remained separated. Hence, THZ1 damages both the nucleolar and Cajal body structures, resulting in their irregular spatiotemporal translocation and colocalization.

To determine whether the translational pathway was specifically affected by THZ1, we stained cells for the presence of DDX21, a nucleolar RNA helicase crucial for translation but not involved in splicing.^21^ DDX21 had distinct nucleolar colocalization with dyskerin (Figure 5f and Supplementary Figure S6c). However, following THZ1 treatment, DDX21 was expelled from the nucleolus and did not translocate to distinct foci (Figure 5f and Supplementary Figure S6c). Instead, it was evenly dispersed throughout the nucleus at a low staining intensity. This demonstrates that the localization of translation-specific proteins such as DDX21 was abrogated, presenting strong evidence that THZ1 disrupts the pathways involved in cytoplasmic translation. This was confirmed by the loss of cytoplasmic translation of nascent proteins following pulse-chase labeling of THZ1-treated cells (Figure 5g). Together, these findings confirm that THZ1 damages the nucleolus and Cajal bodies and heavily disrupts the translational process, resulting in a significant reduction in cytosolic translation.

### THZ1 curtails nuclear speckle formation and organization and results in aberrant mRNA splicing

**Figure 6:**
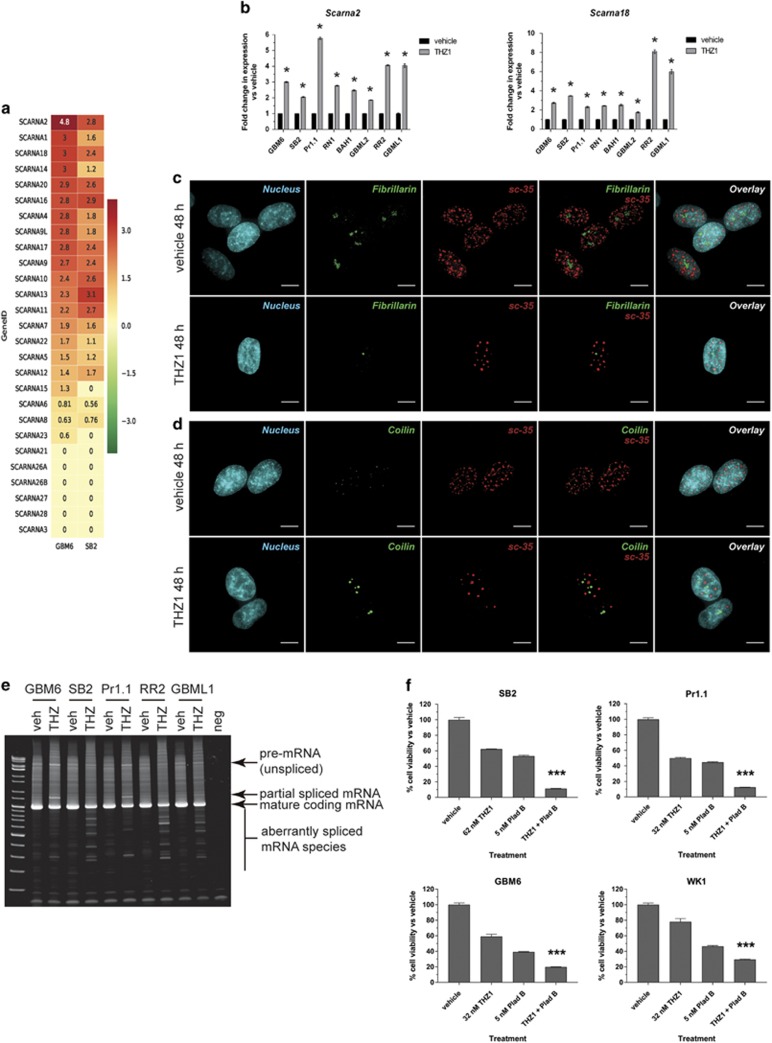
Spliceosomal function is disrupted by THZ1 through nuclear speckle damage, resulting in aberrant mRNA splicing. (**a**) Heat maps documenting the mean log_2_ expression changes of the scaRNA class of non-coding RNAs in THZ1-treated cells vs vehicle controls in GBM6 (left column) and SB2 (right column) as measured by microarray. Green: decrease; red: increase; yellow: no change. (**b**) RT–qPCR validation of upregulation of scaRNA for multiple primary cell lines treated with vehicle or 200 nM THZ1 for 24 h for *Scarna2* (left graph) and *Scarna18* (right graph). All data are graphed as the relative expression of each snoRNA compared with vehicle controls following correction to a multiplexed endogenous *Actb* control±s.e.m. For both graphs, vehicle vs THZ1 *P<0.05 by multiple *t*-test analysis with Holm–Sidak correction, d.f.=4. (**c**, **d**) Immunofluorescence z-stack images for GBM6 cells treated with vehicle or 200 nM THZ1 for 48 h. Images depict the nucleus in blue, sc-35 in red, fibrillarin (**c**) or coilin (**d**) in green and any colocalization of signal in yellow. Scale bar: 10 μm. (**e**) RT–PCR of full-length *EIF4A1* mRNA spanning exon 1 to exon 10 for RNA isolated from multiple primary cell lines treated with vehicle or 200 nM THZ1 for 24 h. Products representing full-length unspliced mRNA, partially spliced mRNA, fully spliced mature mRNA and aberrantly spliced mRNA are indicated. (**f**) HGG cell lines were treated with a combination of low-dose spliceosomal inhibitor, pladienolide B and THZ1. After 7 days, cells were subjected to cell viability luminescence assays. Data are presented as the percentage of viable cells vs vehicle control for each treatment±s.e.m. ***P<0.0001 by one-way analysis of variance (ANOVA) for combination treatments vs all other treatments, d.f.=8.

Our imaging showed that Cajal body formation and distribution were disrupted by THZ1. This was initially puzzling as Cajal bodies are mainly associated with maturation of the spliceosomal RNA before transport to nuclear speckles, not with translation.^22^ Small Cajal body-associated RNAs (scaRNAs) guide small populations of fibrillarin in a manner that post-transcriptionally stabilizes spliceosomal RNA within Cajal bodies.^23^ Further study of the microarray data confirmed that the scaRNA class of small nuclear RNAs was also highly upregulated by THZ1 treatment (Figure 6a). RT–qPCR confirmed this increase for *Scarna2* and *Scarna18* in eight HGG cell lines (Figure 6b).

This led us to believe that not only was the translational apparatus being disrupted but also the spliceosomal apparatus. THZ1 severely disrupted the pattern of sc-35 staining (a non-RNA component of mature spliceosomes, in nuclear speckles), with a large decrease in signal number and a steep increase in focally intense staining in a ‘polka-dot’ pattern (Figure 6c). Strikingly, despite this disruption, the sc-35 staining remained spatiotemporally dissociated from fibrillarin (Figure 6c) and coilin (Figure 6d and Supplementary Figure S7). Interestingly, when THZ1 action was compared with actinomycin D (100 nM) and palbociclib (1 μM) for production of snoRNA and scaRNA, we found that the increase in these non-coding RNAs was specific for THZ1 only after 24 h of drug treatment and was not the result of a generalized transcription or cell cycle inhibition (Supplementary Figures S8a and b). These results show that sites of mature spliceosome function are grossly disrupted but remain spatially dissociated from the fibrillarin/dyskerin/coilin-containing foci after THZ1 treatment.

Multiple snoRNAs can be encoded in separate introns of a host gene, and correct splicing releases equal ratios of snoRNAs.^24^ However, further inspection of the snoRNA expression ratios of genes encoding multiple snoRNAs revealed a profound inequality in snoRNA expression (Figure 5a and Supplementary Tables S2 and S3), suggesting aberrant splicing. To examine this, we conducted RT–PCR on THZ1-treated cells for the full-length snoRNA host genes *Eif4a1* (Figure 6e) and *Rack1* (Supplementary Figure S8c); these assays detect unspliced mRNA, mature spliced mRNA and any partially or aberrantly spliced products. THZ1 treatment led to less mature mRNA and more full-length unspliced mRNA and partially spliced mRNA (Figure 6e and Supplementary Figure S8c). Several products smaller than the mature mRNA were detected in all HGG cell lines after THZ1 treatment, demonstrating aberrant splicing of the mature mRNA product (Figure 6e and Supplementary Figure S8c).

Investigation of RNA surveillance pathways such as nonsense-mediated decay (Supplementary Figure S8d) showed that this regulatory pathway was reduced in response to THZ1, indicating that the partially spliced products may be retained and thereby counter the effects of THZ1. Low THZ1 doses plus the spliceosome inhibitor pladienolide B (which alone inhibited cell growth at low nanomolar concentrations; Supplementary Figure S8e) resulted in supra-additive inhibition of HGG cell line growth (Figure 6f). These results conclusively show that THZ1 has profound effects on the small Cajal body-associated scaRNA population and induces aberrant mRNA splicing by disrupting nuclear speckle formation. These data also identify the spliceosome as a therapeutic vulnerability that can be exploited by combining pladienolide B with THZ1 for supra-additive inhibition of HGG growth.

Finally, to test the suitability of THZ1 as a therapeutic for HGG, we treated mice bearing intracranial GBM6 orthografts with vehicle or THZ1. Our results show no increase in animal survival over 40 days (Supplementary Figure S9), indicating that THZ1 is not crossing the blood–brain barrier and cannot target CDK7 as a naked agent.

## Discussion

We show that CDK7 is crucial for gliomagenesis, confirm that CDK7 is a new target for HGG therapy and provide the first description of the cellular processes affected by the CDK7 inhibitor THZ1, which, cumulatively, result in HGG cell death (Supplementary Figure S10). THZ1 kills HGG cells in a potent manner by initiating multiple convergent cellular insults that abrogate several hallmarks of cancer. Although we have used HGG as a model system here, we found that some of the cellular functions disrupted by THZ1 in HGG are also disrupted in melanoma (Supplementary Figure S11), indicating that our findings may be applicable to other cancers.

HGG invasion, a complicating factor in complete surgical removal of HGG tumors, is decreased by THZ1 treatment. In addition, THZ1 blocks mitotic entry, leading to aneuploidy because of a DNA replication logjam in G2 and DNA damage. This damage is most likely augmented by AIF nuclear translocation because of mitochondrial damage caused by the suppression of nuclear-encoded MRP transcription. An adaptive response to this loss of respiratory equilibrium was observed through upregulation of mitochondrial electron transport chain transcription by POLRMT; ultimately, because of the loss of the MRP translational machinery, this adaptation failed, and the tumor cells succumbed to the drug. As there are no reports of MRP genes being driven off super enhancers,^11, 12, 13, 14, 15^ these studies define a new paradigm of THZ1 action and suggest that eliminating super-enhancer-driven genes is not the only factor contributing to THZ1 action.

THZ1 also combated the HGG-relevant RTK/phosphatidylinositol 3 kinase/mitogen-activated protein kinase pathways,^10^ decreasing the expression of several RTKs (including mutant EGFR) that are targets of multiple small molecule inhibitors and antibodies. THZ1 also deactivated oncogenic pathways harboring several activating mutations in *Pik3ca* and *Kras*, as well as in cells with deletion/inactivation of several tumor suppressors such as *Cdkn2a/b*, *Rb*, *Pten* and *Tp53*. These findings further define a promising therapeutic approach that, instead of targeting these pathways singularly using multiple agents, THZ1 or similar agents may be used to simultaneously negate several active RTK and oncogenic pathways irrespective (at this stage) of differentially altered somatic profiles. This would be of most use in clinical situations where HGG cells from a single tumor have mixed expression of EGFR and its mutants,^25^ where intratumoral regions mosaically express different somatic profiles^1, 2^ or in tumors displaying mixed HGG subtypes, which are associated with poorer prognoses.^26^

Our transcriptome analyses were conducted at a longer time point (24 h) to observe potential adaptation to THZ1. Although, like others,^11, 12, 14, 15^ we observed downregulation of a large proportion of the transcriptome, we also observed upregulation of thousands of transcripts, particularly snoRNA and scaRNA transcripts. We discovered extensive damage to the subnuclear compartments housing these RNAs (the nucleolus, Cajal bodies and nuclear speckles), with irregular spatiotemporal translocation of nucleolar and Cajal body components to identical areas. Some nucleolar disruption has been observed previously with older RNA polymerase II inhibitors such as α-amanitin^27^ but not to the same extent, whereas nucleolar disruption by the intercalating transcription inhibitor actinomycin D leads to spatial separation of key 18S rRNA processing factors and loss of large rRNA precursors,^28^ in agreement with the loss of cytosolic translation observed here.

The spliceosome was also heavily disrupted by THZ1, the first report of a CDK inhibitor doing this, with irregular splicing of the snoRNA host genes, *Eif4a1* and *Rack1* being observed. Spliceostatin A also has similar disruptive effects on nuclear speckles,^29^ supporting our findings that THZ1 abrogates the spliceosome. The improperly spliced transcripts may continue to be utilized given that the RNA surveillance mechanism nonsense-mediated decay, which detects and eliminates these, was downregulated by THZ1. Furthermore, as several integral components of spliceosome maturation colocalized after THZ1 treatment and remained separated from the sc-35-positive nuclear speckles, this may indicate an attempt to counteract the drug by pooling any remaining resources and maintaining spliceosomal function. We exploited this with combination therapy targeting CDK7 and the spliceosome, resulting in a supra-additive reduction in HGG growth. This confirms that attempted retention of spliceosomal function after CDK7 inhibition is a therapeutic vulnerability. In addition, improper splicing and nonsense-mediated decay reduction explain why unequal snoRNA production from host genes housing multiple intronic snoRNA was observed.^24^ This effect required spliceosome disruption and simultaneous transcription and cell cycle inhibition, as it was solely induced by THZ1 after 24 h of drug treatment—in comparison, administration of the general transcription inhibitor actinomycin D and the cell cycle inhibitor palbociclib for the same time period did not result in the increase in these non-coding RNAs. Therefore, we believe that the irregular snoRNA and scaRNA expression ratios may be a candidate biomarker for assessing positive response to THZ1. Such changes can be tracked, as recently demonstrated using qPCR on serum samples from HGG patients to measure changes in circulating non-coding RNAs.^30^

In its current format, THZ1 was unable to cross the blood–brain barrier and provide effective antitumor therapy. Further structural optimization of THZ1 may circumvent this. Such optimization has been successful for EGFR inhibitors modified from an anilinoquinazoline core to a mono-anilino-pyrimidine core, resulting in far superior blood–brain barrier penetration.^31^ In addition, several irreversible CDK7 inhibitors with varying structures have been developed,^32^ which may be more effective at crossing the blood–brain barrier. Other options include using THZ1 in antibody-based conjugates, a strategy that has worked well for glioma in initial clinical trials of ABT-414, an EGFRvIII-specific antibody conjugated to the cytotoxin monomethyl auristatin F.^33^ It will be important to test such possibilities for THZ1, as our data definitively show that if THZ1 can gain access to the tumor, CDK7 inhibition will be a potent and widely effective therapeutic option.

In conclusion, using THZ1 as a model inhibitor, we identified CDK7 as a promising target for HGG therapy and uncovered the mechanism by which THZ1 kills cells: a cascade of cellular perturbations resulting in DNA damage, mitochondrial damage, translation loss and spliceosome malfunction. A potential combination therapy to augment THZ1 activity was identified, as well as an increase in snoRNA and/or scaRNA as a possible biomarker of a positive response to THZ1 treatment. We also discover that THZ1, in its current format, does not cross the blood–brain barrier and hence will require reformatting for use as an HGG therapeutic. Ultimately, this work may lead to a far more effective therapeutic countermeasure to eliminate HGG, a devastating and incurable disease.

## Materials and methods

### Cell lines

Primary patient-derived neurosphere cell lines were isolated from surgically resected patient tissue and established as previously described.^17^ Patient tumor tissue was collected after obtaining informed consent and with human ethics approval from the QIMR Berghofer Medical Research Institute and Royal Brisbane and Women’s Hospital human research ethics committees. All cell lines were maintained in StemPro medium (Life Technologies, Waltham, MA, USA) consisting of Dulbecco’s modified Eagle’s medium/F12 knock out medium, 100 U penicillin/streptomycin, 2 mM GlutaMAX, neurobasal supplement, 10 ng/ml basic fibroblast growth factor and 10 ng/ml EGF, at 37 °C and 5% CO_2_. All cells were used at low passage for a maximum of 8 weeks. Once per week, cells were dissociated with Accutase (Life Technologies) and passaged.

The melanoma cell lines A375, A2058, IPC-298, SK-MEL-28, CHL-1 and MeWo were kindly donated by Professor Grant McArthur and Dr Karen Sheppard from the Peter McCallum Cancer Centre (Melbourne, Australia). These cell lines were maintained in RPMI-1640 medium containing 10% fetal bovine serum, 20 mM HEPES and 2 mM GlutaMAX at 37 °C and 5% CO_2._

The BJ fibroblast line (a kind gift from Professor Jus St John, Hudson Institute of Medical Research) and HS5 bone marrow stromal cell line (a kind gift from Dr Ashish Banerjee, Hudson Institute of Medical Research), as well as the SF767 glioma cell line, were maintained in Dulbecco’s modified Eagle’s medium/F12 medium containing 10% fetal bovine serum and 2 mM GlutaMAX at 37 °C and 5% CO_2_.

### Antibodies and reagents

All primary and secondary antibodies used in this study, including information about the target, species, dilutions and sources, are documented in Supplementary Table S4. THZ1 was purchased from MedChem Express (Monmouth Junction, NJ, USA); palbociclib, from Selleck Chemicals (Houston, TX, USA); Caspase 3/7 Glo assays, from Promega (Madison, WI, USA); and ViaLight proliferation assays, from Lonza (Basel, Switzerland). Annexin V FACS kits, FxCycle PI/RNase staining solution, TaqMan pre-optimized RT–qPCR gene expression assays, Superscript III reverse transcription kits, Platinum PCR SuperMix, SYBR Safe DNA stain and Diamond antifade mounting medium with DAPI were purchased from Life Technologies. RNA miniprep kits and DNAse kits were purchased from Qiagen (Venlo, Netherlands); Matrigel, from Corning (Corning, NY, USA); emetine and actinomycin D, from Sigma Aldrich (St Louis, MO, USA); and EXPRESS ^35^S Protein Labeling Mix, from Perkin Elmer (Waltham, MA, USA).

### Proliferation assays

THZ1 was titrated from 1 μM in log_2_ decrements and added to cells. Owing to the slower proliferative rate of primary GBM cell lines,^17^ we incubated cells for 7 days with the drug to allow the testing of the effect of drugs in this system over a similar number of doubling times to that for conventional, fetal bovine serum based, cell lines used in other laboratories. After 7 days, ViaLight luminescence-based proliferation assays were performed as per the manufacturer’s instructions.

### RT–qPCR and alternative splicing RT–PCR

Total RNA (1.5 μg) was reverse transcribed using the Superscript III reverse transcription kit. For qPCR, 1 μl complementary DNA was amplified in triplicate using 2 × gene expression master mix (Life Technologies) and pre-optimized 6-carboxyfluorescein (FAM)-labeled TaqMan assays targeting *Mrps6*, *Mrpl17*, *Polrmt*, *Mt-*c*o1*, *Mt-cyb*, *Egfr*, *EgfrvIII*, *Pdgfra*, *Snora65*, *Snord95*, *Scarna2* or *Scarna18* in multiplex with a VIC-labeled *Actb* TaqMan assay on a QuantStudio 6 real time PCR machine (Life Technologies). The 2^−ΔΔCt^ method was used to compare relative gene expression of THZ1-treated samples to vehicle controls after normalization to endogenous *Actb*.

To capture alternatively spliced mRNA products or full-length unspliced mRNA, primers targeting exon 1 (forward primer 5′-ATGTCTGCGAGCCAGGATTCCCG-3′) and exon 10 (reverse primer 5′-CTGCTGCACATCAATGCCTCTGGC-3′) of *Eif4a1* and exon 1 (forward primer 5′-CCGCCATGACTGAGCAGATGACCC-3′) and exon 8 (reverse primer 5′- CGTGTGCCAATGGTCACCTGCC-3′) of *Rack1* were designed. Conventional PCR was conducted using 1.5 μl complementary DNA, 1 μM each primer and Platinum PCR SuperMix with the following parameters: hotstart (95 °C, 3 min); 35 cycles of 95 °C, 30-s denaturation, 65 °C, 30-s annealing and 72 °C, 10-min extension; followed by 4 °C hold. Product was separated at 150 V on 4–20% Tris/boric acid/EDTA buffer polyacrylamide gels and visualized using SYBR Safe staining on an ultraviolet transilluminator with an orange filter.

### Flow cytometry

Cell-surface RTK FACS was conducted as previously described^34^ (primary and secondary antibodies, Supplementary Table S4) on a FACS CANTO II machine using FlowJo software (FlowJo, Ashland, OR, USA). Annexin V analyses were conducted as per the manufacturer’s instructions. For cell cycle analyses, cells were processed and stained using FxCycle PI/RNase (Life Technologies) as per the manufacturer’s instructions.

### Immunoprecipitation and western blotting

Treated cells were washed in ice-cold phosphate-buffered saline before lysis in RIPA buffer (Thermo Scientific, Waltham, MA, USA) supplemented with 2 × Halt single use dual phosphatase/protease inhibitor cocktail (Thermo Scientific, Waltham, MA, USA), 5 mM EDTA and 2 mM activated Na_3_VO_4_. To analyze UPF1 function, 1.25 mg total lysate, diluted to 1 ml in TXC buffer^35^ was mixed with antibody for 2 h at 4 °C with rotation and incubated overnight with protein A/G agarose beads (4 °C with rotation). For all whole-cell-lysate tests, 50 μg total protein was analyzed. All samples were separated on 4–12% Bis-Tris gradient sodium dodecyl sulfate–polyacrylamide gel electrophoresis gels, transferred to polyvinylidene difluoride membranes using an iBlot apparatus (Thermo Scientific), probed overnight at 4 °C with rocking using primary antibody (Supplementary Table S4) diluted in Odyssey buffer containing 0.1% Tween-20, and detected using secondary antibodies (Supplementary Table S4). The infrared Odyssey scanner was used on the 700 and 800 nm channels, and images were processed using Odyssey software (LI-COR, Lincoln, NE, USA).

### Three-dimensional spheroid invasion assay

Spheroid invasion assays were conducted as previously described^36^ on cells treated with dimethylsulfoxide vehicle or 200 nM THZ1 for 48 h.

### Caspase activation assays

Cleaved caspase 3/7 was measured using the Caspase 3/7 Glo assay as per the manufacturer’s instructions.

### Microarray and bioinformatics

RNA extracted from GBM6 or SB2 cells treated for 24 h in quadruplicate was hybridized to SurePrint G3 Human v2 8 × 60K microarrays (Agilent, Santa Clara, CA, USA) as per the manufacturer’s instructions. All arrays were pre-processed using the R package *limma*.^37^ The normalized and filtered data from 19 155 probe sets having a variation coefficient >0.05 across all samples were tested for significant differential expression between the THZ1 and vehicle groups using the *limma* package^37^ from Bioconductor (Roswell Park Cancer Institute, Buffalo, NY, USA). A linear model was fitted to the (log_2_) normalized expression data, and moderated *t*-statistics were computed and adjusted for multiple testing using the Benjamini and Hochberg method.

### Immunofluorescence

Immunofluorescence was conducted essentially as previously described^38^ with a permeabilization/blocking step after fixation (1 h at 22 °C in Odyssey buffer containing 0.3% Triton X-100 and 0.1% Tween-20 (OTT)). Antibodies (Supplementary Table S4) were diluted in OTT. Images were acquired at × 100 equivalent magnification using × 60 augmented magnification and a DeltaVision deconvolution microscope (GE Healthcare, Pittsburgh, PA, USA). Z-stack images were analyzed using ImageJ (National Institutes of Health, Bethesda, MD, USA).

### *Ex vivo* HGG tissue treatment and analysis

Organotypic culture was performed as previously described^39^ using a de-identified patient specimen obtained with the above permissions. After treatment, the sample was fixed in 10% neutral buffered formalin and embedded in paraffin. For immunohistochemistry, 4 μm sections were probed with an anti-pSer139 γH2AX rabbit monoclonal antibody (Supplementary Table S4) at 1:500, developed with diaminobenzidine chromagen and counterstained with hematoxylin. Images were acquired with an Aperio AT-Turbo (Leica, Wetzlar, Germany) at × 20 magnification.

### High-resolution respirometry

Fifty (50) μl containing 2 × 10^6^ cells treated with vehicle or 200 nM THZ1 for 24 h was injected into sealed 2 ml chambers containing oxygenated StemPro medium in an Oxygraph-2k machine (OROBOROS, Innsbruck, Austria) with baseline oxygen concentration and oxygen flux rates already established. Ten microliters of dimethylsulfoxide vehicle or THZ1 (200 nM final concentration in the chamber) was injected after 5 min, to continue drug selection. The oxygen concentration in the isolated chambers (nmol/ml) and the oxygen consumption rate (as oxygen flux per volume (in pmol/(s × ml))) were measured every 1.8 s over several hours using OROBOROS Oxygraph software. Raw data were exported to Excel and analyzed using Prism software (GraphPad, La Jolla, CA, USA).

### Pulse-chase radiolabeling of newly synthesized protein

Cells grown to 80% confluence were treated with dimethylsulfoxide vehicle or 200 nM THZ1 for 24 h. Media were removed and replaced with methionine- and cysteine-free Dulbecco’s modified Eagle’s medium for 30 min. For mitochondrion-specific translation, emetine (100 μg/ml final concentration) was added to block cytosolic translation. Cells were pulsed with 0.05 mCi ^35^S-labeled methionine and cysteine for 2 h before cell lysis and separation by 4–12% sodium dodecyl sulfate–polyacrylamide gel electrophoresis. Newly synthesized protein was detected following exposure of radiolabeled gels to a phosphor-screen using a GE Amersham Typhoon 9400 variable mode imager (GE Healthcare).

### Intracranial orthografts

Animal experiments were approved by the QIMR Berghofer animal ethics committee (project P1572). Intracranial orthograft experiments were essentially as previously described^34^ using the previously described dosing schedule^14^ via intraperitoneal administration. This study was in accordance with the Australian Code of Practice for the Care and Use of Animals for Scientific Purposes (2004).

### Statistics

All data were analyzed using Prism 5.02 software (GraphPad). Student’s *t*-test was used for pairwise analyses, whereas a one-way analysis of variance was used for grouped analyses within a normal distribution with appropriate *post hoc* tests. Standard error was assigned to all data where applicable. Significance was attributed when *P*<0.05.

### Data availability

All microarray data have been deposited in the Gene Expression Omnibus (GEO) database under accession number GSE94931.
