# Supplementary material for: Cyclin-dependent kinase 7 is a therapeutic target in high-grade glioma
Source: Oncogenesis. 2017 May 15;6(5):e336–. doi: 10.1038/oncsis.2017.33 (PMC5523066; doi:10.1038/oncsis.2017.33)
Supplement: Supplementary Figures and Tables [file oncsis201733x1.pdf]

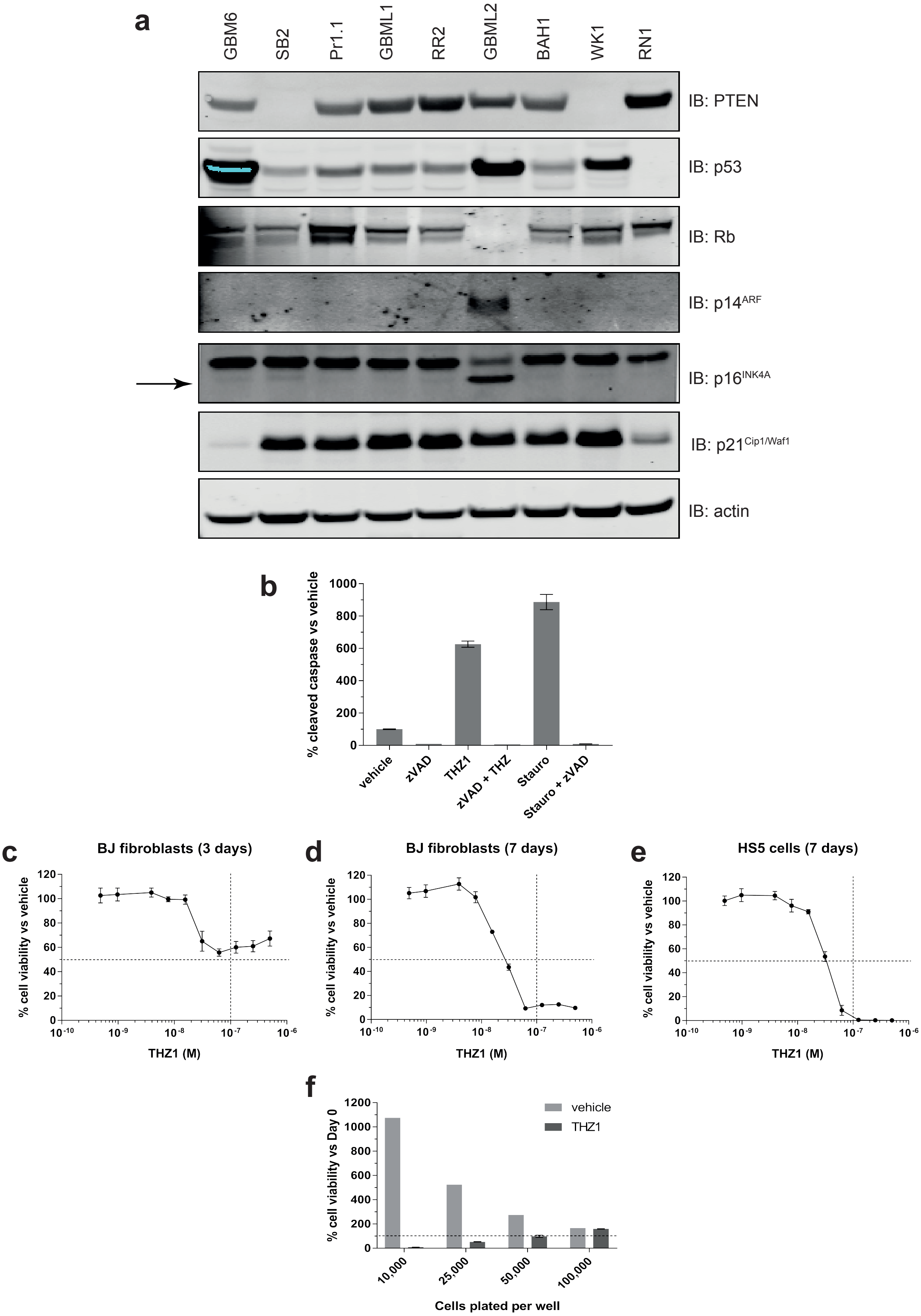

Supplementary Fig 1 Initial characterisation of the response of cancerous and normal cells to THZ1. **(a)** Tumour suppressor and oncogene characterisation in patient-derived, HGG primary cell line panel. Cells were harvested and lysates analysed by western blotting. Actin (bottom panel) was used as a loading control. The arrow indicates the p16-specific band. (**b**) Caspase 3/7 activity assay confirming that the pan caspase inhibitor, z-VAD-fmk, abolishes THZ1 and staurosporine induced caspase activity in RR2 HGG cells. (**c-d**) Proliferation curves for BJ Fibroblasts treated for (**c**) 3 days or (**d**) 7 days with 200 nM THZ1. (**e**) Proliferation curves for HS5 bone marrow stromal cells treated for 7 days with 200 nM THZ1. For all proliferation curves, data is represented as the percentage of viable cells vs vehicle control at each dose ± s.e.m. Horizontal dashed line = 50% inhibition of cell viability. Vertical dashed line; 100 nM THZ1 concentration. (**f**) Cell viability graphs for SF767 HGG cells plated at various confluencies and treated for 7 days with 200 nM THZ1. Dashed line indicates the same reading as plating at day 0.

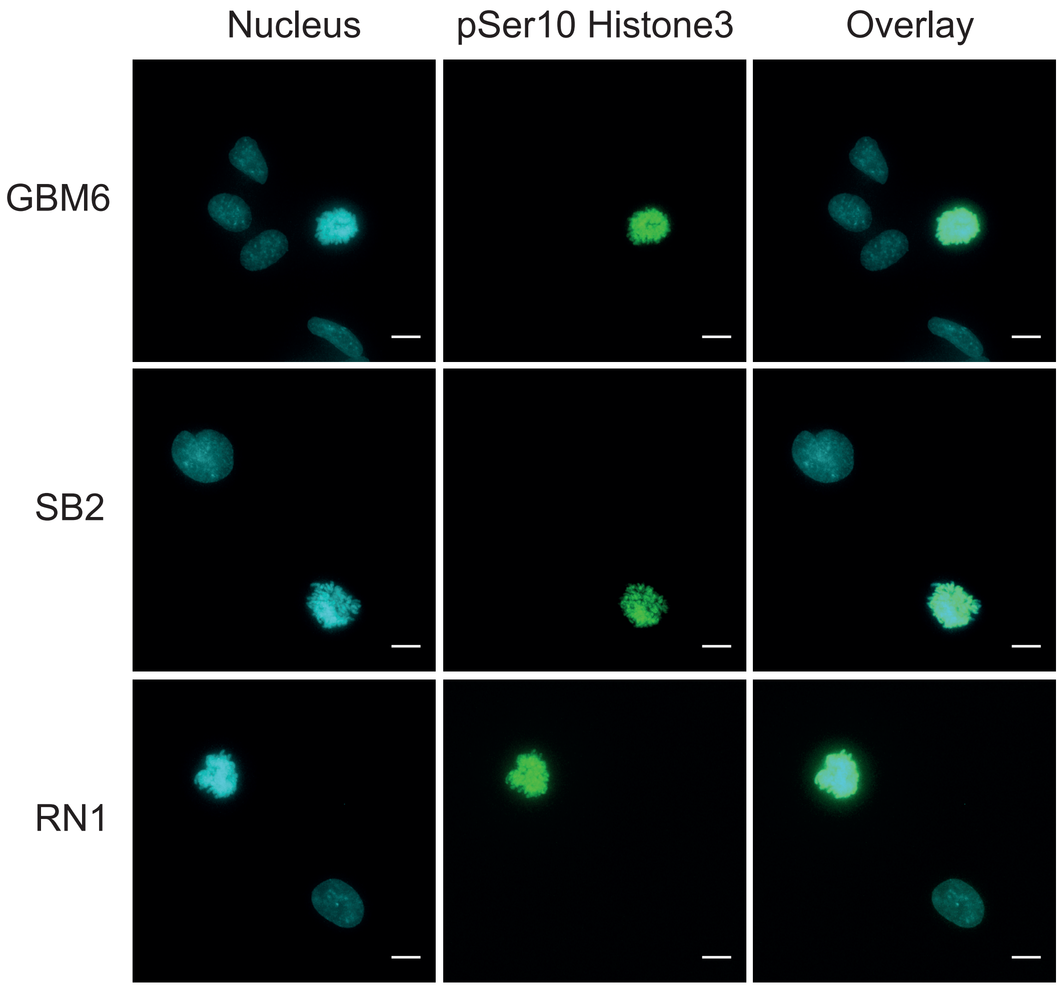

Supplementary Fig 2 Confirmation by immunofluorescence that pSer10 Histone3 only stains mitotic HGG cells. Cells were grown on chamber slides for 48 h before fixation, permeablisation and staining for pSer10 Histone3 (green). Nucleus = blue. Scale bar = 10 μm. Note that only mitotic cells demonstrating chromosome condensation are bound by the antibody.

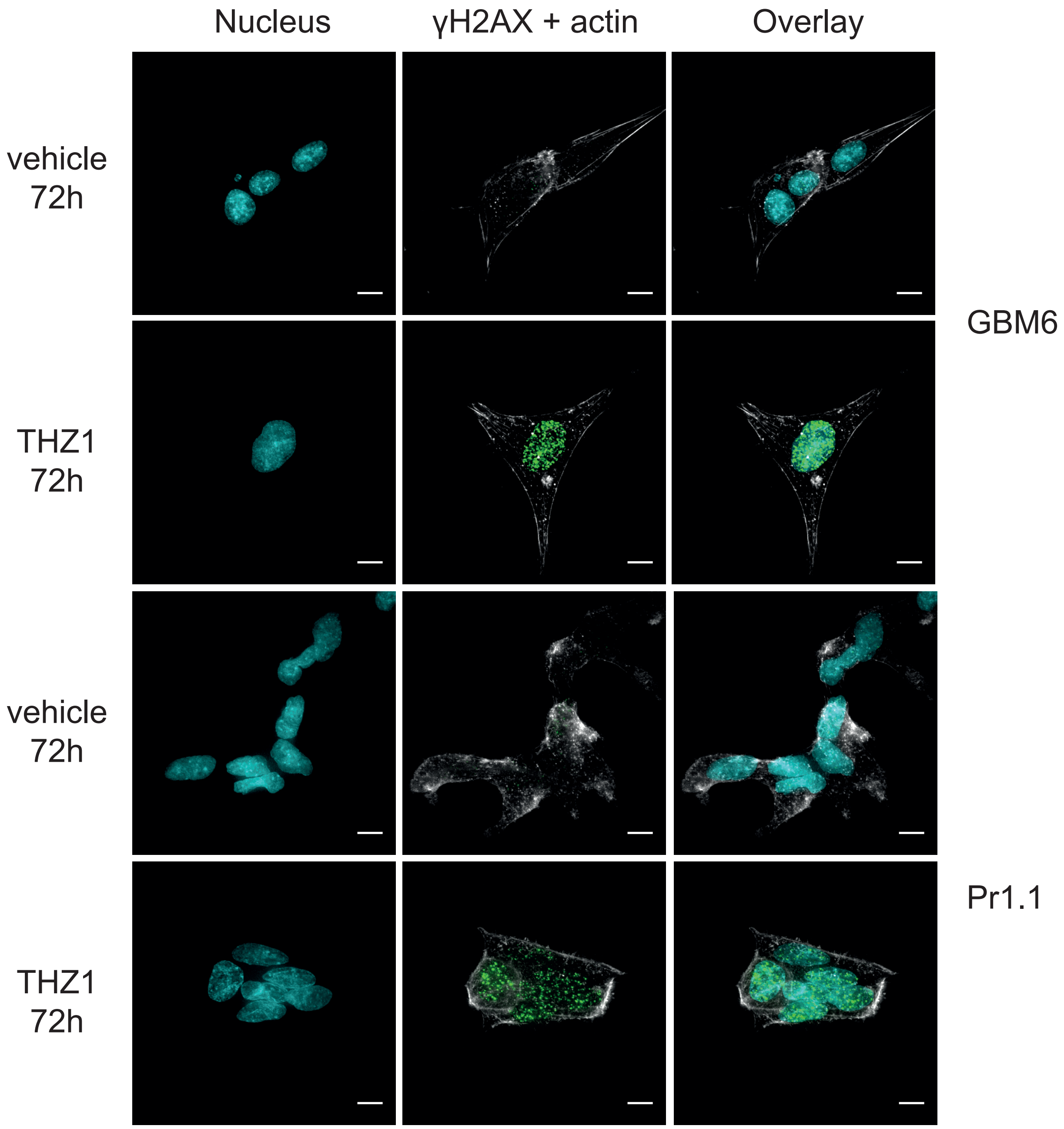

Supplementary Fig 3 THZ1 induces DNA damage in GBM6 and Pr1.1 cell lines. Cells were treated for 72 h with vehicle or 200 nM THZ1, fixed, permeablised and stained for pSer139 γH2AX (green). Cells were also co-stained for actin (grey) and nucleus (blue). Scale bar = 10 μm.

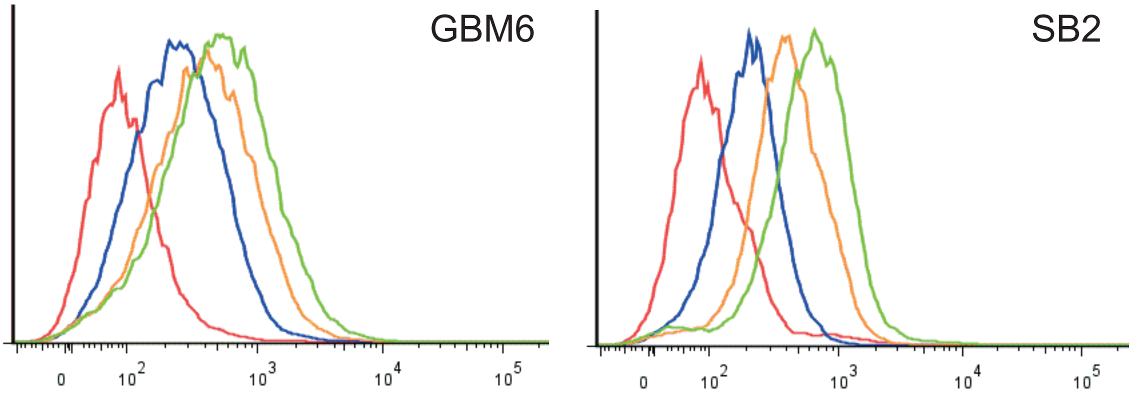

Supplementary Fig 4 THZ1 induces cumulative DNA damage over time. Cells were treated with vehicle or 200 nM THZ1 for 24 h, 48 h or 72 h and then processed for intranuclear staining of γH2AX by flow cytometry. The signal for γH2AX detection steadily increases at each time point for THZ1 treated cells. Red = vehicle control; blue = THZ1 24 h; orange = THZ1 48 h; green = THZ1 72 h.

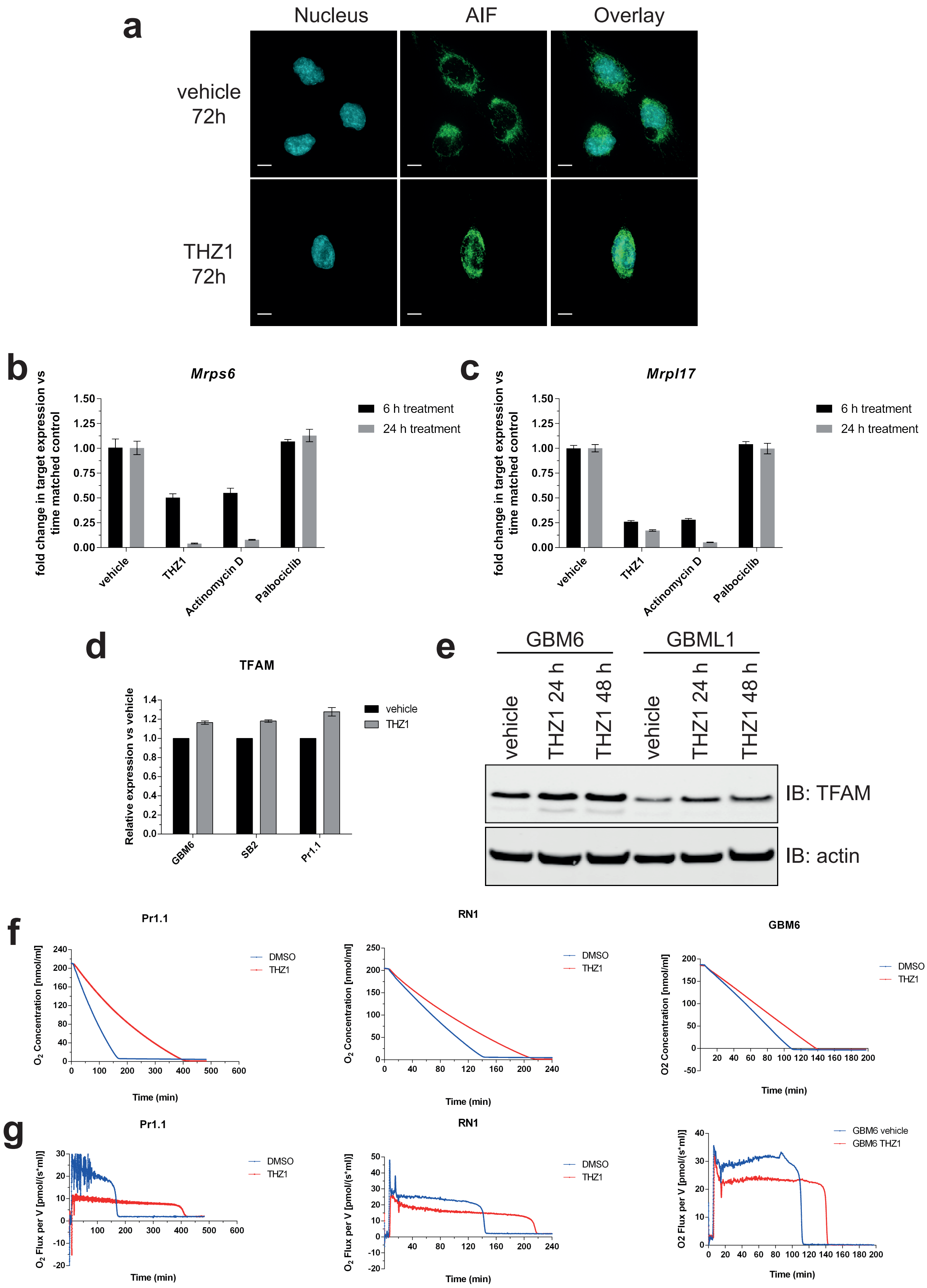

Supplementary Fig 5 THZ1 results in mitochondrial damage. (**a**) AIF (green) translocation to the nucleus (blue) in SB2 cells treated for 72 h with vehicle or 200 nM THZ1. Scale bar = 10 µm. RT-qPCR profiles for *Mrps6* (**b**) or *Mrpl17* (**c**) detection following various treatments at 6 h or 24 h in RR2 cells. (**d**) TFAM RT-qPCR profiles after treatment of cells with vehicle or 200 nM THZ1 for 24 h. (**e**) TFAM western blots from lysates isolated from cells treated with vehicle or 200 nM THZ1 for 24 h and 48 h. (**f-g**) THZ1 suppresses oxidative respiration in Pr1.1, RN1 and GBM6 cells. Cells were treated with vehicle or 200 nM THZ1 for 24 h after which they were tested by high resolution respirometry. (**f**) Oxygen concentration in each sealed chamber over time. (**g**) Oxygen flux for each chamber over time.

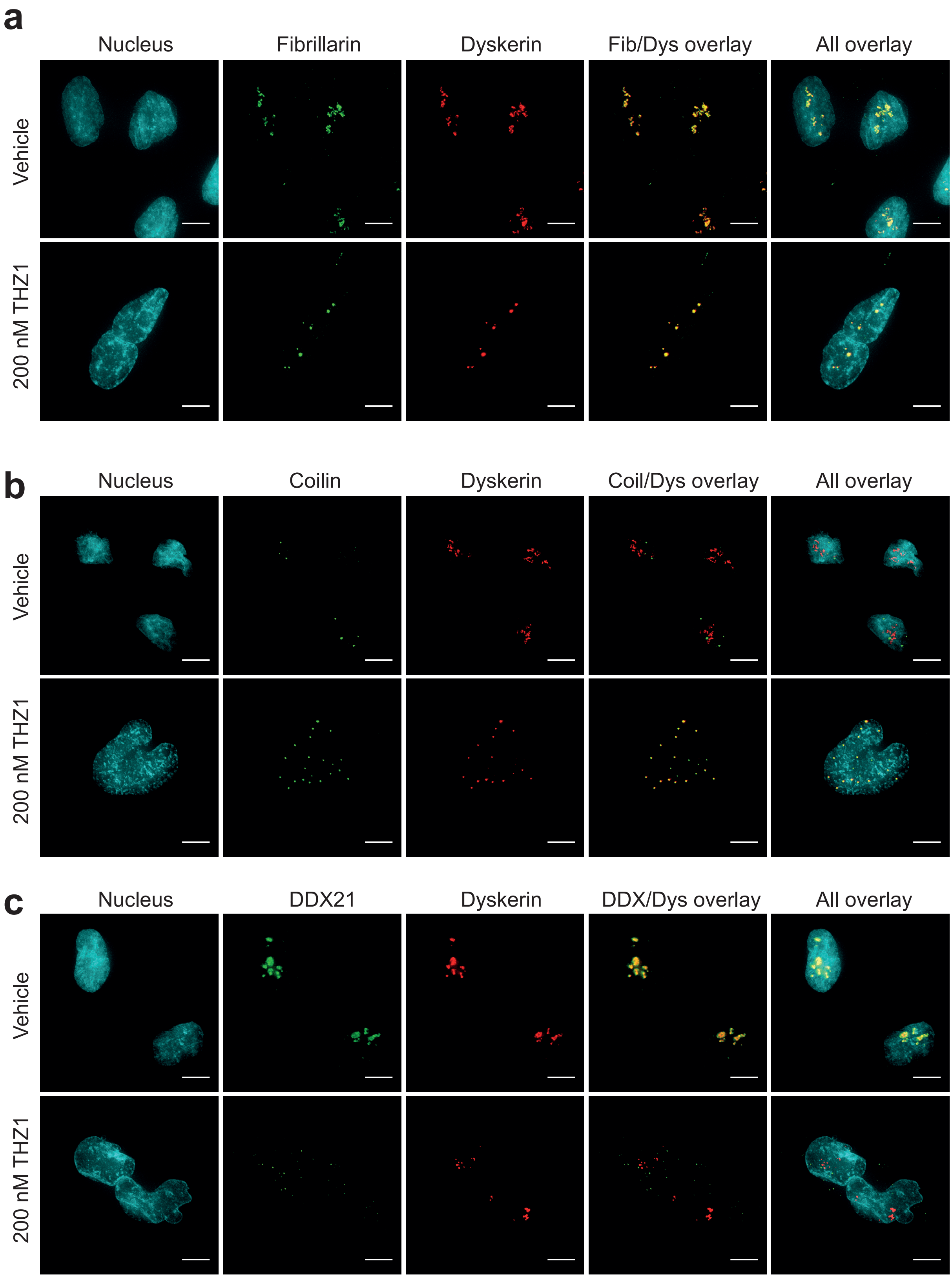

Supplementary Fig 6 THZ1 disrupts the nucleolus and Cajal bodies in SB2 cells, leading to unusual spatiotemporal association of coilin with the nucleolar components. Cells were treated for 48 h then stained for immunofluorescence for the nucleolus using dyskerin and fibrillarin (**a**); the Cajal bodies and nucleolus using coilin and dyskerin (**b**) or the nucleolus using DDX21 and fibrillarin (**c**). Nucleus = blue, co-localisation = yellow. Scale bar = 10 μm.

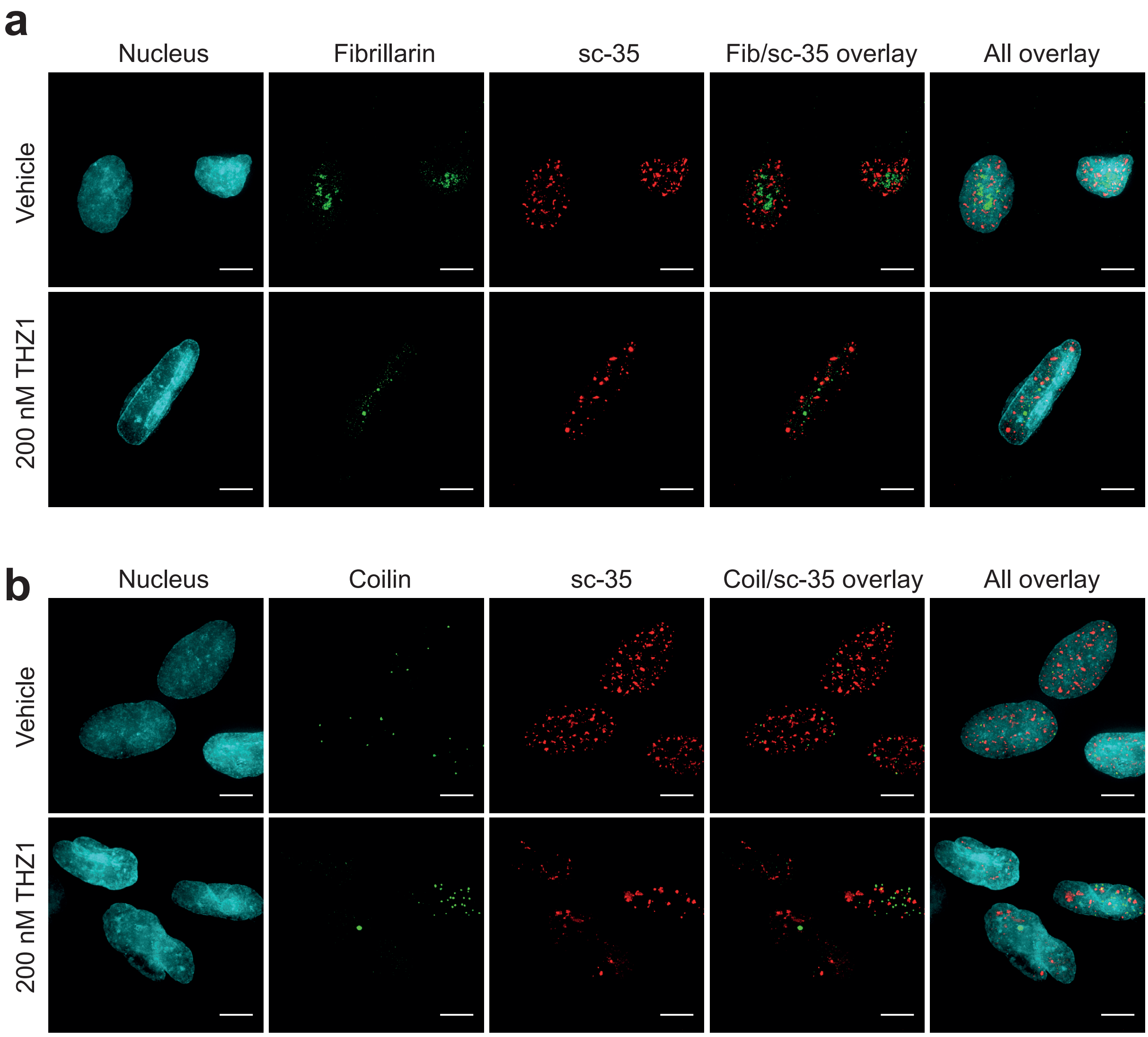

Supplementary Fig 7 THZ1 disrupts the nuclear speckles, the sites of mature spliceosome function in SB2 cells. Cells were treated for 48 h then stained for immunofluorescence for the nucleolus and nuclear speckles using fibrillarin and sc-35 (**a**) or the Cajalbodies and nuclear speckles using coilin and sc-35 (**b**). Nucleus = blue, co-localisation = yellow. Scale bar = 10 μm.

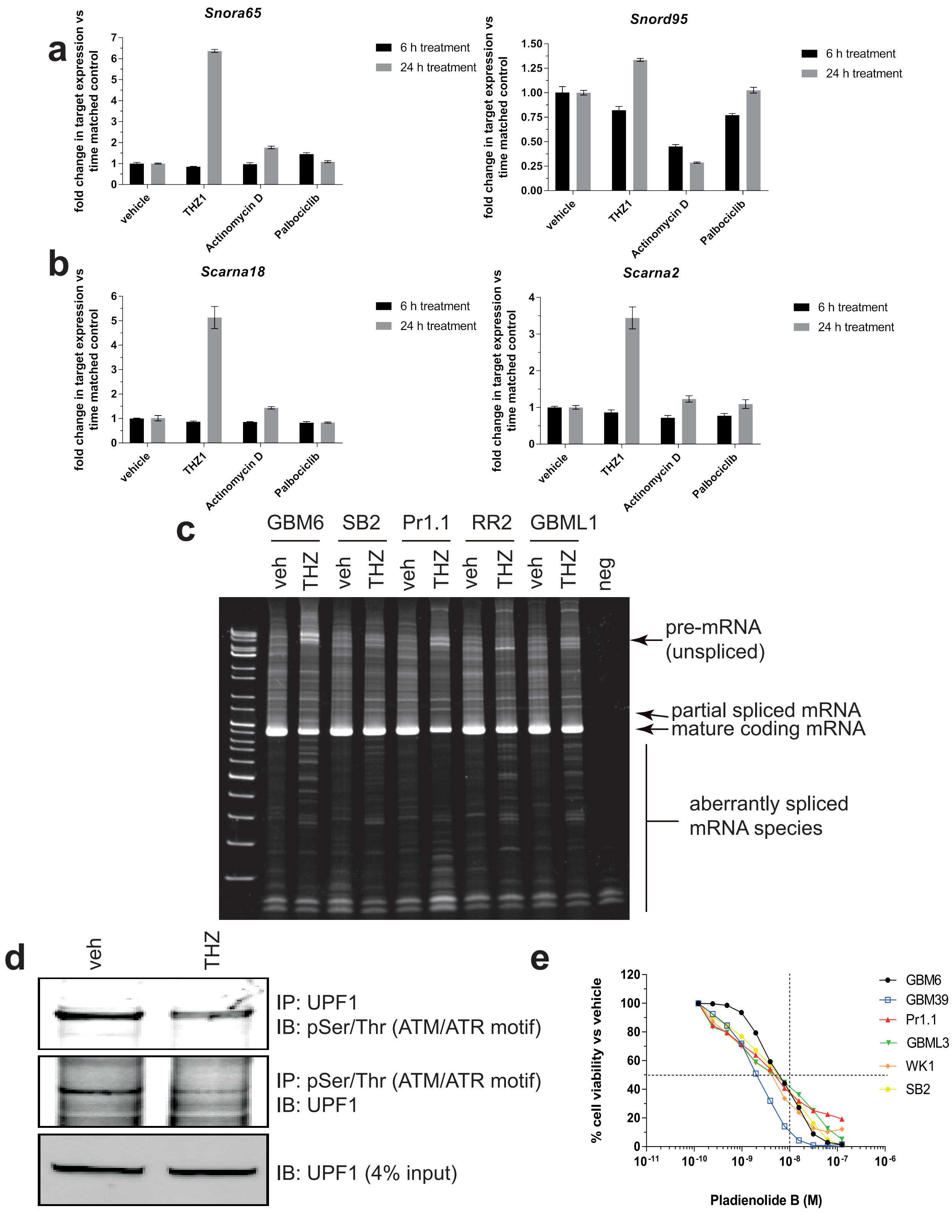

Supplementary Fig 8 THZ1 peturbs spliceosomal function. RT-qPCR profiles for *Snora65* and *Snord95* (**a**) or *Scarna18* and *Scarna2* (**b**) following treatment of RR2 HGG cells with various drugs for 6 h or 24 h. (**c**) RT-PCR for the entire length of the snoRNA host gene, *Rack1*, demonstrating dysfunctional transcript splicing. (**d**) NMD is downregulated in response to THZ1 in GBM6 cells. Immunoprecipitation and western analysis of phosphorylated UPF1 and total UPF1 was conducted on cell lysates isolated after treatment with vehicle or 200 nM THZ1. A western blot for total UPF1 conducted on 4% of the IP lysate was used as an input control. (**e**) Primary HGG cells are inhibited by low nanomolar concentrations of the spliceosome inhibitor, Pladienolide B. HGG cells were treated with a titration of Pladienolide B for 7 days and subjected to cell viability assays. Data are presented as the percentage of viable cells vs vehicle control at each dose ± s.e.m. Horizontal dashed line = 50% inhibition of cell viability. Vertical dashed line; 10 nM Pladienolide B concentration.

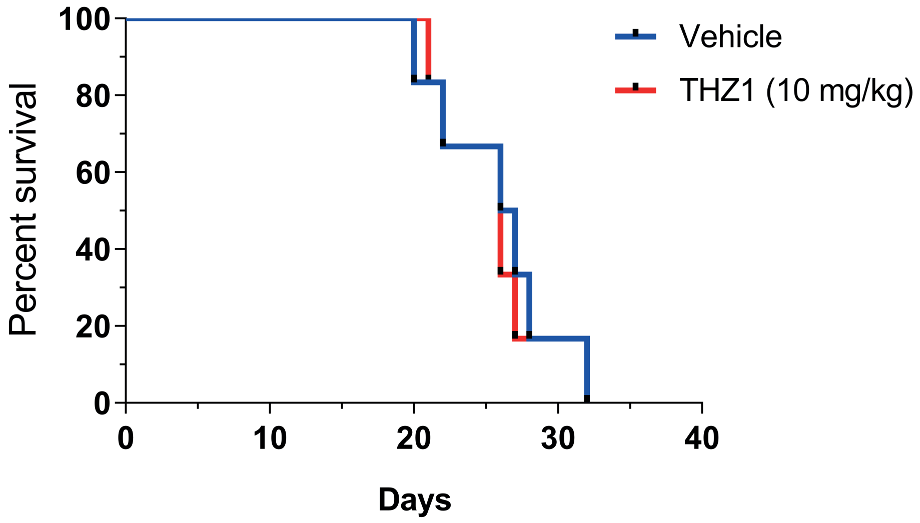

Supplementary Fig 9 THZ1 does not cross the blood brain barrier. Nude mice were implanted intracranially with GBM6 cells before treatment with vehicle or 10 mg/kg THZ1 q2d for two weeks. Data are presented as Kaplan-Meier curves demonstrating percentage survival of mice over time.

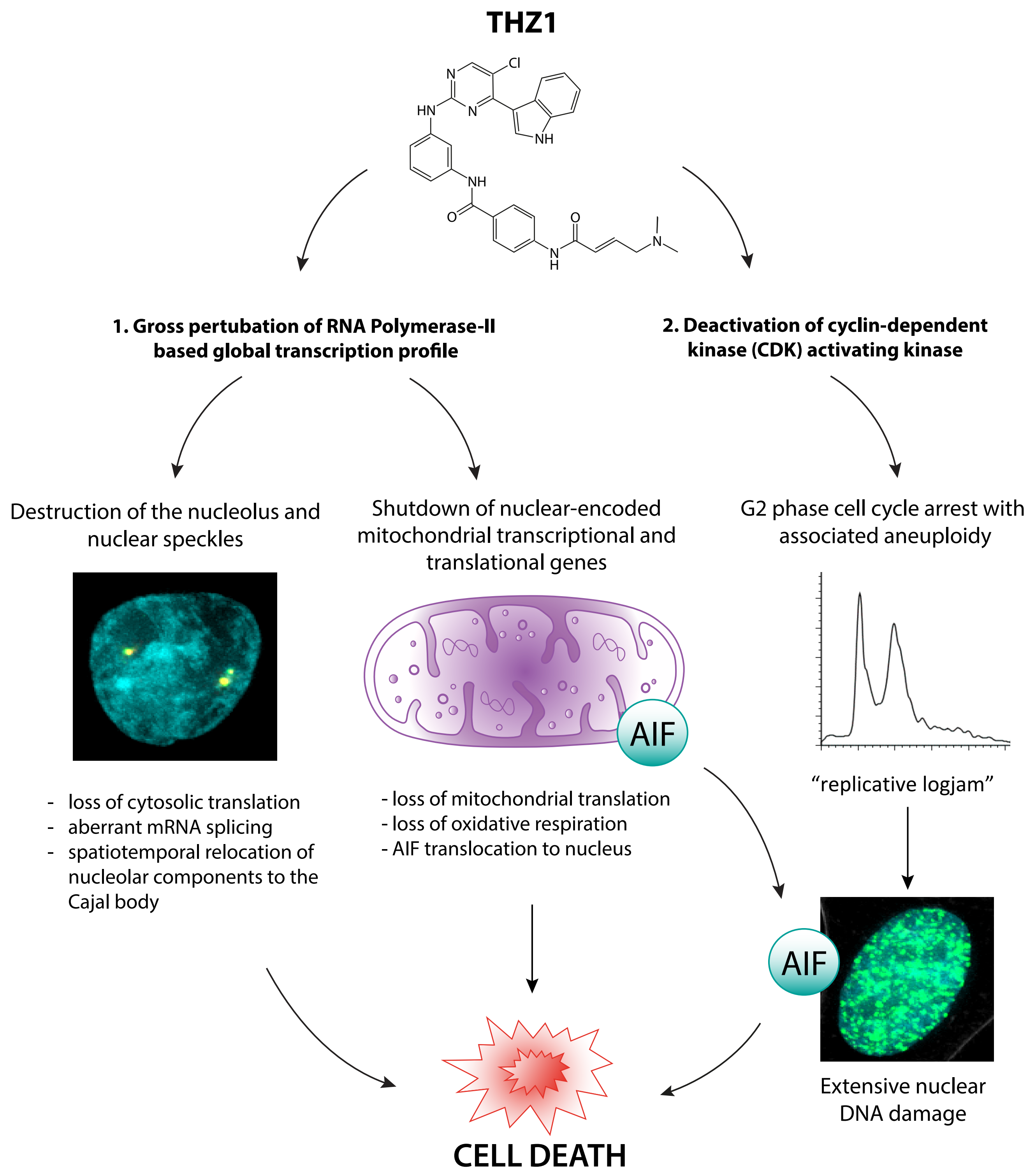

Supplementary Fig. 10 Overview of THZ1 mechanism of action in primary HGG cells. THZ1 initiates a cascade of cellular perturbations ultimately leading to mitochondrial damage, DNA damage, loss of cytoplasmic translation and spliceosome malfunction, leading to HGG cell death.

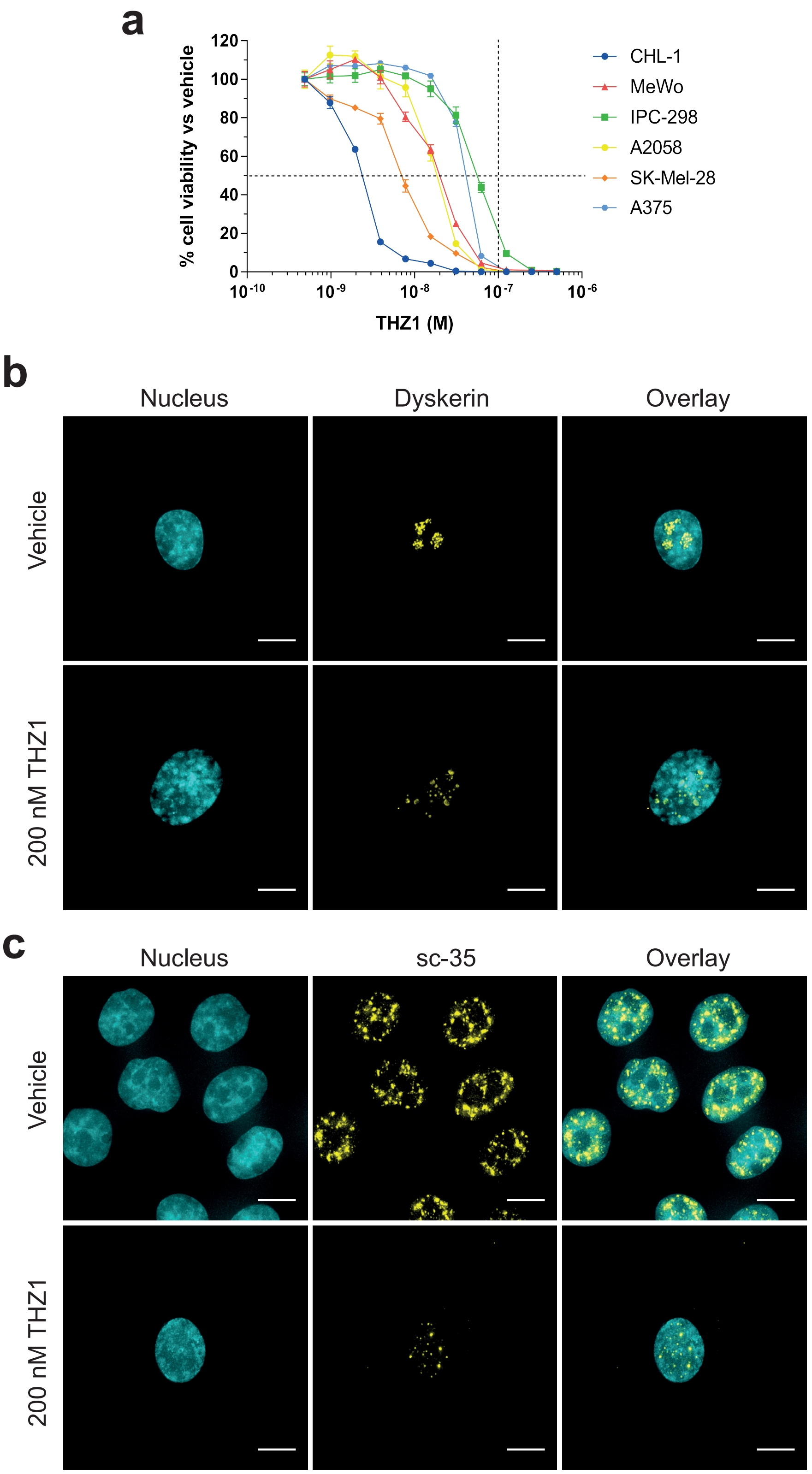

Supplementary Fig 11 THZ1 has a similar mechanism of action in melanoma cells. (**a**) Cell viability curves for several melanoma cell lines, representing a range of different RAF, RAS and NF1 genotypes, treated with titrated THZ1 for 5 days. Horizontal dashed line: IC_{50}; vertical dashed line: 100 nM THZ1. The nucleolus (**b**) and nuclear speckles (**c**) are disrupted by THZ1 treatment in the melanoma cell line A375. Cells were stained by immunofluorescence for dyskerin (nucleolus) and sc-35 (nuclear speckles). Nucleus = blue, Target = yellow. Scale bar = 10 μm.

Supplementary Table 1 Known subtypes and exome changes for neurosphere panel used in this study

		**Known gene mutation status**											
**Cell line**	**Subtype**	***CDKN2A***	***CDKN2B***	***P53***	***RB***	***EGFR***	***MET***	***PTEN***	***PIK3CA***	***PIK3R1***	***RAS***	***NF1***	***MDM2***
GBM-L1	?	Δ/Δ	Δ/Δ			wt/wt					wt/G13D	wt/G1977V	
GBM-L2	?	wt/wt	wt/wt	wt/P146S	exon 17 c.1499 G>A splice acceptor site	wt/wt							
GBM-L3	?	?	?			wt/wt					wt/G13D		
GBM6	Classical	Δ/Δ	Δ/Δ	R273H/R273H		EGFRvIII (amp)							
BAH1	Neural	Δ/Δ	Δ/Δ			EGFRvIII (amp)				Δ/Δ			
SB2	Classical	Δ/Δ	Δ/Δ			wt/A289V/H304Y (amp)	C800F/C800F	Δ/Δ	Y1021H/Y1021H				W329C/W329C
WK1	Mesenchymal	Δ/Δ	Δ/Δ	Δ/Δ		wt/wt		Δ/Δ					
RR2	?	Δ/Δ	Δ/Δ			wt/wt		G132D/G132D				C1016Y/C1016Y	
RN1	Classical	Δ/Δ	Δ/Δ	Δ/Δ		wt/wt							
GBM39	Classical	Δ/Δ	Δ/Δ			EGFRvIII (amp)							
Pr1.1	?	Δ/Δ	Δ/Δ			wt/H773_V774insPH							

Supplementary Table 4 Primary and Secondary antibodies and their applications as used in this study

PRIMARY ANTIBODIES					
**Antibody target**	**Clone**	**Species/clonality**	**Supplier**	**Catalogue number**	**Application and dilution**
Actin (pan)	ACTN05 (C4)	mouse mAb	Thermo Scientific	MA5-11869	Western blot - 1:10 000
AIF	D39D2	rabbit mAb	Cell Signaling	5318	Immunofluorescence - 1:300
Akt (pan)	40D4	mouse mAb	Cell Signaling	2920	Western blot - 1:1000
Akt (pSer473)	D9E	rabbit mAb	Cell Signaling	4060	Western blot - 1:1000
CDK-1	POH1	mouse mAb	Cell Signaling	9116	Western blot - 1:1000
CDK-1 (pThr161)	-	rabbit polyclonal	Cell Signaling	9114	Western blot - 1:1000
CDK-2	D-12	mouse mAb	Santa Cruz Biotechnology	sc-6248	Western blot - 1:500
CDK-2 (pThr160)	-	rabbit polyclonal	Cell Signaling	2561	Western blot - 1:1000
CDK-6	1F9.1	mouse mAb	Millipore	MABC280	Western blot - 1:1000
CDK-6 (pTyr24)	-	rabbit polyclonal	Santa Cruz Biotechnology	sc-293097	Western blot - 1:500
CDK-7	C-19	rabbit polyclonal	Santa Cruz Biotechnology	sc-529	Western blot - 1:500
CDK-7 (pThr170)	-	rabbit polyclonal	Santa Cruz Biotechnology	sc-130185	Western blot - 1:500
Coilin	D2L3J	rabbit mAb	Cell Signaling	14168	Immunofluorescence - 1:300
DDX21	-	rabbit polyclonal	NovusBIO	NBP1-83310	Immunofluorescence - 1:200
Dyskerin	H-3	mouse mAb	Santa Cruz Biotechnology	sc-373956	Immunofluorescence - 1:100

EGFR	mAb806	mouse mAb	Abbot Pharmaceuticals	N/A	Western blot - 1:5000; Flow cytometry - 1:1000
EGFR (panitumumab)	-	human mAb	Amgen	N/A	Flow cytometry - 1:4000
ERK 1/2	L34F12	mouse mAb	Cell Signaling	4696	Western blot - 1:1000
ERK 1/2 (pThr202/pTyr204)	D13.14.4E	rabbit mAb	Cell Signaling	4370	Western blot - 1:1000
FGFR3	C-15	rabbit polyclonal	Santa Cruz Biotechnology	sc-123	Western blot - 1:500
FGFR3	MM0279-6G11	mouse mAb	Abcam	ab89660	Flow cytometry - 1:250
Fibrillarin	C13C3	rabbit mAb	Cell Signaling	2639	Immunofluorescence - 1:200
Histone H3 (pSer10) - AF647 conjugate	D2C8	rabbit mAb	Cell Signaling	3458	Flow cytometry - 1:50; Immunofluorescence - 1:400
Histone γ-H2AX (pSer139)	20E4	rabbit mAb	Cell Signaling	9718	Immunohistochemistry - 1:500
Histone γ-H2AX (pSer139) - AF647 conjugate	20E4	rabbit mAb	Cell Signaling	9720	Flow cytometry - 1:50; Immunofluorescence - 1:50
MET	LMH85	mouse mAb	Hudson Institute	N/A	Flow cytometry - 1:1000
MRP-S6	E-8	mouse mAb	Santa Cruz Biotechnology	sc-390597	Western blot - 1:500
p14	4C6/4	mouse mAb	Cell Signaling	2407	Western blot - 1:1000
p21 (Waf1/Cip1)	12D1	rabbit mAb	Cell Signaling	2947	Western blot - 1:1000

p53	DO-1	mouse mAb	Santa Cruz Biotechnology	sc-126	Western blot - 1:1000
PDGFR-α	16A1	mouse mAb	Abcam	ab96569	Flow cytometry - 1:250
PDGFR-α	D1E1E	rabbit mAb	Cell Signaling	3174	Western blot - 1:1000
PDGFR-β	2B3	mouse mAb	Cell Signaling	3175	Western blot - 1:1000
pSer/Thr (ATM/ATR motif) MultimAb mix	-	rabbit mAb	Cell Signaling	6966	Western blot - 1:1000; Immunoprecipitation - 1:100
PTEN	D4.3	rabbit mAb	Cell Signaling	9188	Western blot - 1:1000
Rb	4H1	mouse mAb	Cell Signaling	9309	Western blot - 1:1000
RNA Polymerase II CTD (pSer2)	3E10	rat mAb	Millipore	04-1571	Western blot - 1:500
sc-35	-	mouse mAb	Abcam	ab11826	Immunofluorescence - 1:500
STAT3	79D7	rabbit mAb	Cell Signaling	4904	Western blot - 1:1000
STAT3 (pTyr705)	3E2	mouse mAb	Cell Signaling	9138	Western blot - 1:1000
TFAM	D5C8	rabbit mAb	Cell Signaling	8076	Western blot - 1:1000
UPF1	D15G6	rabbit mAb	Cell Signaling	12040	Western blot - 1:1000; Immunoprecipitation - 1:50

SECONDARY ANTIBODIES					
**Antibody target**	**Fluorophore Conjugate**	**Host**	**Supplier**	**Catalogue number**	**Application and dilution**
human IgG	Alexa Fluor 647	goat	Life Technologies	A21445	Flow cytometry - 1:300
mouse IgG	Alexa Fluor 647	goat	Life Technologies	A21235	Flow cytometry - 1:300; Immunofluorescence - 1:100
mouse IgG	Alexa Fluor 647	goat	Life Technologies	A21058	Western blot - 1:10 000
rabbit IgG	Alexa Fluor 488	goat	Life Technologies	A11034	Flow cytometry - 1:300; Immunofluorescence - 1:100
rabbit IgG	IRDye 800	goat	Licor Biosciences	926-32211	Western blot - 1:2000
rat IgG	IRDye 800	goat	Licor Biosciences	926-32219	Western blot - 1:2000
